# Spatial profiling of the mouse colonic immune landscape associated with colitis and sex

**DOI:** 10.1038/s42003-024-07276-1

**Published:** 2024-11-29

**Authors:** Matilda Holm, Lina Stepanauskaitė, Anna Bäckström, Madeleine Birgersson, Fabio Socciarelli, Amena Archer, Charlotte Stadler, Cecilia Williams

**Affiliations:** 1 SciLifeLab, Department of Protein Science, KTH Royal Institute of Technology, Solna, Sweden; 2 Division of Biosciences and Nutrition, Department of Medicine Huddinge, Karolinska Institutet, Huddinge, Sweden; 3 Department of Pathology and Laboratory Medicine, Weill Cornell Medicine, New York, NY USA

**Keywords:** Imaging the immune system, Colon, Inflammation

## Abstract

Inflammatory intestinal conditions are a major disease burden. Numerous factors shape the distribution of immune cells in the colon, but a spatial characterization of the homeostatic and inflamed colonic immune microenvironment is lacking. Here, we use the COMET platform for multiplex immunofluorescence to profile the infiltration of nine immune cell populations in mice of both sexes (*N* = 16) with full spatial context, including in regions of squamous metaplasia. Unsupervised clustering, neighborhood analysis, and manual quantification along the proximal-distal axis characterized the colonic immune landscape, quantified cell-cell interactions, and revealed sex differences. The distal colon was the most affected region during colitis, which was pronounced in males, who exhibited a sex-dependent increase of B cells and reduction of M2-like macrophages. Regions of squamous metaplasia exhibited strong infiltration of numerous immune cell populations, especially in males. Females exhibited more helper T cells and neutrophils at homeostasis and increased M2-like macrophage infiltration in the mid-colon upon colitis. Sex differences were corroborated by plasma cytokine profiles. Our results provide a foundation for future studies of inflammatory intestinal conditions.

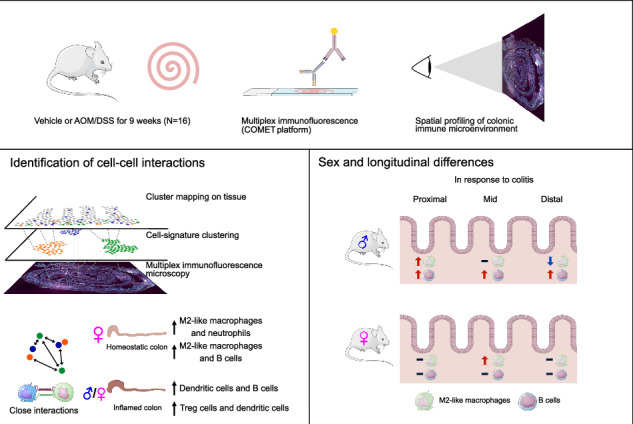

## Abstract (web summary)

Spatial profiling of the colonic immune microenvironment at homeostasis and during colitis in mice, with a focus on sex differences in immune infiltration.

## Introduction

Inflammatory bowel disease (IBD) is a major public health burden, with nearly 5 million cases diagnosed worldwide, and the incidence is increasing in most countries^1^. Moreover, chronic inflammation drives the development and progression of many types of diseases, including colorectal cancer (CRC)^2^. Patients with autoimmune-related IBD, which includes ulcerative colitis and Crohn’s disease, have a well-documented increased risk of CRC^3,4^. Immune cells in the microenvironment secrete pro-inflammatory cytokines, chemokines, and growth factors, which induce proliferation and contribute to tumor initiation and progression^5^. Various types of immune cells accumulate at sites of intestinal inflammation^6^, and the depletion of dendritic cells (DCs) ameliorates colitis in mice^7,8^. Macrophages^9,10^, helper T (Th) cells^11^, and neutrophils^12^ that infiltrate and accumulate in the intestinal mucosa promote intestinal inflammation through the secretion of pro-inflammatory cytokines such as IL-17 (IL17), TNF-α (TNF)^13^, and IFNγ (IFNG), which can in turn influence macrophage activation^14^. Cytotoxic T (Tc) cells, which secrete pro-inflammatory cytokines, are also implicated in the pathogenesis and progression of IBD^15^. Anti-inflammatory (M2-like) macrophages and regulatory T (Treg) cells, on the other hand, antagonize colonic inflammation and promote healing^16^. Further, the accumulation of B cells in the inflamed intestine appears to suppress mucosal inflammation, as documented in IBD patients^17^.

Notably, sex differences are apparent in both IBD and CRC. Women are more likely to develop Crohn’s disease whereas more men develop ulcerative colitis, especially after the age of 50. The global number of disability-adjusted life-years is higher among females than among males^1^, while men with IBD have a higher risk of developing CRC as a result than women with IBD do^18^. Men also have an earlier age of onset of CRC in general and a 31% higher incidence rate than women^19,20^. Differences in tumor location, molecular alterations, and survival have also been reported^21^, and we have previously identified different candidate diagnostic and prognostic markers for CRC patients depending on sex^22^. The cause for these sex differences is not fully established but is attributed to lifestyle, expression of X and Y-linked genes, and sex hormones^23,24^. Sex differences in intestinal immune cell accumulation have not been well studied. However, it was recently reported that female patients had higher numbers of T cells in the colon compared to males, although the study did not distinguish between Th and Tc cells^25^. Overall, studies have mostly profiled the colonic infiltration of one or a few different immune cell populations without investigating differences in spatial distribution or between sexes. Recent studies have used techniques such as RNA sequencing and flow cytometry of lesions to map gene expression at the single-cell level at homeostasis^26^ and during colitis to identify mucosal and immune signatures of IBD and pathways involved in these diseases^27–30^. In blood samples, natural killer (NK) and Treg cell numbers, as well as CD8^+^ T cell counts, have been shown to be higher in men, while CD4^+^ T cell counts and B cell numbers were higher in women^31^. However, the colonic immune landscape has not been profiled with regard to spatial context and sex differences, neither in humans nor mice.

Comprehensive studies are challenging in humans as the whole, healthy colon is, on average, 150 cm long and 6 cm wide and is rarely resected, excluding comprehensive spatial molecular analysis. Additionally, lifestyle differences between women and men (such as diet and alcohol consumption) influence the colon and are difficult to separate from inherent biological sex differences. The mouse, in contrast, can be held in controlled environments, eliminating differences in diet and exposure to various inflammatory compounds. The murine colon is ~13 cm in length and 4 mm in width, enabling complete spatial analysis within a histological section of a Swiss-rolled colon. Treating mice with azoxymethane (AOM) and dextran sulfate sodium (DSS) induces colitis in a controlled manner that accurately mimics the multistep development of colitis-associated colorectal cancer, with very similar features to human CRC also at the molecular level^32^. AOM/DSS treatment increases the numbers of infiltrating macrophages, T cells^33^, neutrophils^34^, NK cells^35^, and DCs^36^ in the colon, which contribute to disruption of the epithelial barrier^37^. Further, B cells have been shown to be the dominant immune cell type in the colon during the recovery phase in a mouse model of DSS-induced colitis^17^. Similarly, macrophages^38^, T cells^39^, neutrophils^40^, NK cells^41^, DCs^6^, and B cells^42^ are known to infiltrate and accumulate in the intestine of IBD patients. A study by Parigi et al. used spatial transcriptomics to profile the transcriptomic landscape of the mouse intestine (female mice only, control *n* = 1, DSS-treated *n* = 2) and observed distinct molecular regionalization of the colon during steady-state conditions and found that the distal colon was the most affected at the histological and transcriptomic levels^43^. Using the AOM/DSS model, we and others have previously shown that numerous sex differences in CRC development also exist in mice: males develop larger tumors compared to females^23^ and exhibit a stronger colonic transcriptomic response to colitis. In particular, the colonic gene expression of pro-inflammatory cytokines (e.g., *Tnfα (Tnf)*, *Il6*, and *Il1b)* was increased in males compared to females^44^. We have also observed clear sex differences in the colon transcriptome of healthy mice (fed a control diet) regarding genes involved in immune response, cell proliferation, and canonical Wnt signaling, and in the composition of the caecal microbiome^45,46^. When mice were fed a high-fat diet, sex differences in the corresponding infiltration of F4/80^+^ macrophages were noted^46^. While sex differences exist in the colon and in the immunological response to colitis, they have not been thoroughly characterized.

In this study, we used the novel COMET platform (Lunaphore Technologies) for multiplex immunofluorescence analysis of Swiss-rolled colon sections from wild-type mice of both sexes to characterize the infiltration of 9 immune cell populations at homeostasis and during colitis with full spatial and longitudinal context. We applied unsupervised clustering followed by neighborhood analysis to characterize the response of the colonic immune cell populations to colitis, both in terms of altered numbers and cell-cell interactions between different populations. This indicated significant reductions of M2-like macrophages and increases of neutrophils, macrophages/Th cells, and B cells, along with increased interactions between Tc cells (with DC cells, neutrophils, and B cells) and neutrophils (with DC cells, Treg and Tc cells, and B cells). Only females exhibited close interactions between neutrophils and M2-like macrophages at homeostasis. Manual quantifications of infiltration along the proximal-distal axis showed that females exhibited a gradient of elevated numbers of neutrophils towards the distal end and of Th cells towards the proximal end, whereas males exhibited reduced B cells towards the distal end at homeostasis. The distal colon was the most affected region during colitis, which was pronounced in males, who exhibited a sex-dependent increase of B cells and reduction of M2-like macrophages. Females, on the other hand, displayed increased M2-like macrophage infiltration in the mid-colon during colitis. Regions of squamous metaplasia exhibited strong infiltration of multiple immune cell populations, with males exhibiting significantly higher NK cell and neutrophil infiltration in this region compared to the rest of the colon. Plasma cytokine profiles further indicated sex differences in the response to colitis. Our work contributes to defining the spatial profile of the intestinal immune landscape and provides a background for studies of intestinal conditions.

## Results

**Fig. 1:**
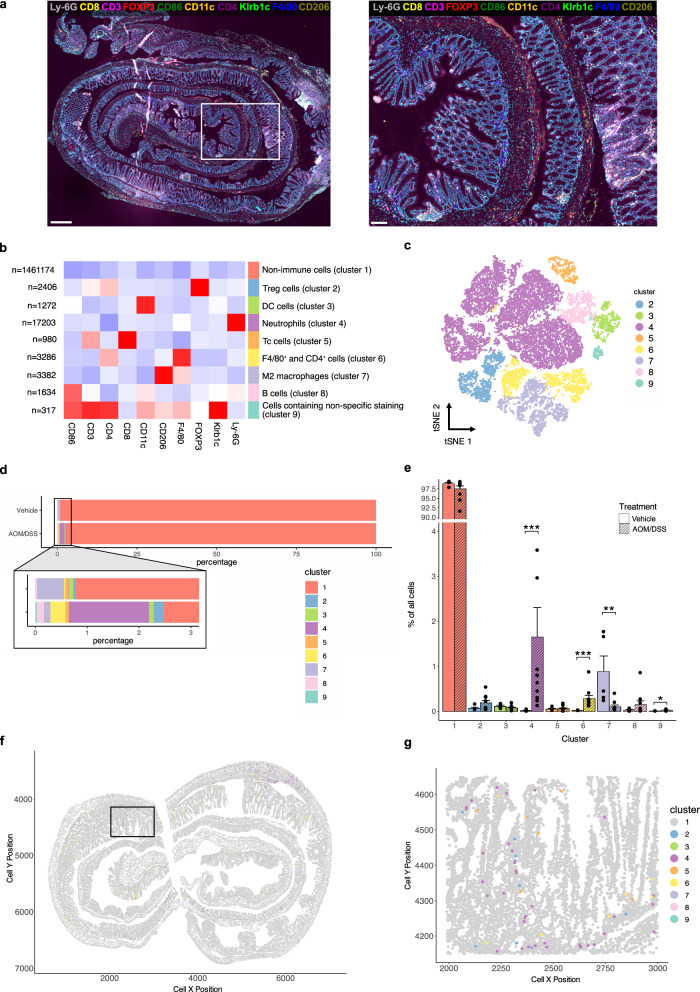
The immune microenvironment of the healthy and inflamed colon characterized by COMET multiplex immunofluorescence and unsupervised clustering analysis. **a** Profiling of 10 markers in an AOM/DSS-treated colon stained using the COMET platform. Ly-6G (gray), CD8 (yellow), CD3 (magenta), FOXP3 (red), CD86 (green), CD11c (orange), CD4 (purple), Klrb1c (lime), F4/80 (blue), and CD206 (olive). Scale bars: 500 μm (left) and 100 μm (right). **b** Heatmap generated from unsupervised clustering performed on staining intensities of all cells counted in all samples. The number of cells in each cluster is shown on the left. **c** tSNE mapping of clusters 2–9 (non-immune cells excluded). **d** The percentage of cells in clusters 1–9 in all samples grouped by treatment. Vehicle-treated: top. AOM/DSS-treated: bottom. **e** The percentage of cells in clusters 1–9 from (**d**) grouped separately by cluster and treatment. Results are given as mean ± SEM and statistical analysis was performed using Mann–Whitney tests (**e**). **f**, **g** The spatial distribution of cells in clusters 1–9 in an AOM/DSS-treated colon sample.

To profile and visualize colonic immune cell distribution and infiltration, we used the COMET platform to perform multiplex immunofluorescence staining of Swiss-rolled colons from vehicle-treated (*n* = 3 males and *n* = 3 females) and AOM/DSS-treated (*n* = 5 males and *n* = 5 females) mice (representative images of H&E-stained vehicle- and AOM/DSS-treated samples are given in Supplementary Fig. 1a, b). Crypt proliferation, extent of colitis, and disease activity index were previously evaluated in these mice^23^. We stained for a panel of 10 markers (Table 1 and Supplementary Fig. 1c), optimized and designed to allow simultaneous profiling of 9 different immune cell populations with full spatial context (Fig. 1a). For each sample, areas with nonspecific staining, dust, edge effect, or areas that were torn were manually excluded. All cells within the remaining annotated areas were counted and intensity data for each cell and marker was extracted using QuPath.

**Table 1:** Antibodies used for multiplex immunofluorescence on the COMET platform

Antibody	Catalog number	Lot number	Host species (M/P^a^)	Provider	RRID	Concentration μg/mL (undiluted)	Dilution	Marker of	Gene	QuPath classifier measurement
Anti-CD3	ab5690	GR3356033-6	Rabbit (P)	Abcam	AB_305055	200	1:250	T cells	*Cd3e*	Cytoplasm: Mean
CD4 (4SM95) eBioscience	14-9766-82	2335913	Rat (M)	Thermo Fisher	AB_2573008	500	1:50	Th cells (with CD3)	*Cd4*	Cytoplasm: Mean
CD8a (4SM15) eBioscience	14-0808-82	2384000	Rat (M)	Thermo Fisher	AB_2572861	500	1:50	Tc cells (with CD3)	*Cd8a*	Cell: Mean
FOXP3 (FJK-16s) eBioscience	14-5773-82	2397986	Rat (M)	Thermo Fisher	AB_467576	500	1:50	Treg cells	*Foxp3*	Nucleus: Mean
F4/80 (D2S9R) XP	70076	5	Rabbit (M)	CST	AB_2799771	435	1:100	Macrophages	*Adgre1*	Cytoplasm: Mean
CD86 (E5W6H)	19589	5	Rabbit (M)	CST	AB_2892094	550	1:200	M1-like macrophages (with F4/80) and (CD86)^b^	*Cd86*	Cytoplasm: Mean
CD206/MRC1 (E6T5J) XP	24595	3	Rabbit (M)	CST	AB_2892682	93	1:400	M2-like macrophages (with F4/80)	*Mrc1*	Cytoplasm: Mean
Klrb1c/CD161c (E6Y9G)	39197	1	Rabbit (M)	CST	AB_2892989	50	1:50	NK cells	*Klrb1c*	Cytoplasm: Mean
Ly-6G (E6Z1T)	87048	4	Rabbit (M)	CST	AB_2909808	68	1:50	Neutrophils and PMN-MDSCs	*Ly6g*	Nucleus: Max
CD11c (D1V9Y)	97585	1	Rabbit (M)	CST	AB_2800282	25	1:100	DCs	*Itgax*	Cell: Mean

^a^*M* indicates monoclonal, *P* polyclonal.

^b^To obtain a population enriched in activated B cells, CD86^+^ cells were quantified by excluding CD86^+^F4/80^+^ cells, CD86^+^CD11c^+^ cells, and CD86^+^F4/80^+^CD11c^+^ cells.

### Unsupervised clustering and mapping characterize the colitis-induced immune landscape

To obtain an overview of the distribution of immune cells present, unsupervised clustering analysis was performed using the staining intensities of all counted cells and markers in all samples (Fig. 1b). The largest cluster represented non-immune cells (cluster 1: 1.46 M cells), as expected. t-distributed stochastic neighbor embedding (tSNE) mapping of the eight immune clusters (including cluster 9) showed that the clusters clearly separated (Fig. 1c). The clusters corresponded to regulatory T cells (Treg cells, cluster 2), CD11c^+^ cells (assumed to be enriched for DCs and referred to as such in the remainder of the text, cluster 3), Ly-6G^+^ cells (assumed to be enriched for neutrophils and subsequently referred to as such, cluster 4), cytotoxic T cells (Tc cells, cluster 5), M2-like macrophages (cluster 7), and CD86^+^ cells (assumed to be enriched for B cells and referred to as such, cluster 8). One cluster, cluster 6, contained cells expressing F4/80 (pan-macrophage marker) and CD4 (Th cell marker), while the smallest cluster, cluster 9, included cells with high intensities of multiple markers. Manual inspection of cells in cluster 6 indicated that the cells that were considered positive for both markers were, in fact, not double-positive cells but instead F4/80^+^ cells in proximity to CD4^+^ cells. Further, manual inspection of cluster 9 indicated incidences of non-specific staining in multiple channels (Supplementary Fig. 2). We observed increases in the numbers of immune cells in most clusters following AOM/DSS treatment (Fig. 1d, e), with significant increases in the numbers of neutrophils (cluster 4), F4/80^+^ and CD4^+^ cells (cluster 6), and cells displaying non-specific staining (cluster 9). Further, the numbers of anti-inflammatory M2-like macrophages (cluster 7) decreased significantly during colitis, and no significant changes were noted in the numbers of Treg (cluster 2), DCs (cluster 3) and Tc cells (cluster 5). Thus, unsupervised clustering of the COMET staining largely generated clear immune cell profiles aligning with the expected populations.

### The spatial distribution and cell interactions of infiltrating immune cell populations

**Fig. 2:**
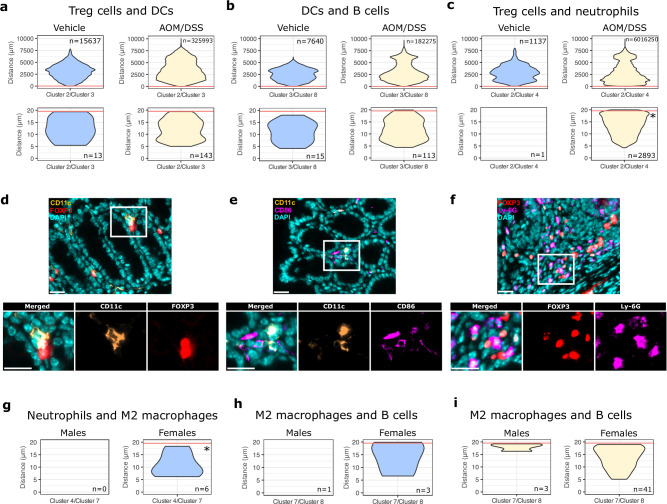
The spatial immune landscape of the colon, including interactions between immune cells and sex differences, as characterized by SPIAT. **a**–**c** Violin plots showing the distribution of pairwise distances between (**a**) Treg cells (cluster 2) and DCs (cluster 3), (**b**) DCs and B cells (cluster 8), and (**c**) Treg cells and neutrophils (cluster 4). Vehicle-treated samples (blue, left panels), AOM/DSS-treated samples (yellow, right panels), all distances (top panels), 0–20 μm (bottom panels). **d**–**f** Images of close interactions identified in (**a**–**c**): (**d**) Treg cells (cluster 2, red) and DCs (cluster 3, orange), (**e**) DCs and B cells (cluster 8, magenta), and (**f**) Treg cells and neutrophils (cluster 4, magenta). Scale bars: 20 μm (top) and 10 μm (bottom). **g**–**i** Violin plots showing the distribution of pairwise distances in males and females between (**g**) Neutrophils (cluster 4) and M2-like macrophages (cluster 7) in vehicle-treated samples, and M2-like macrophages and B cells (cluster 8) in (**h**) vehicle-treated samples and (**i**) AOM/DSS-treated samples. * indicates *P* < 0.05.

Immune cells communicate and interact with each other through both direct and indirect mechanisms. They can recruit, induce, activate, and suppress each other, affecting their overall spatial organization. To visualize such distributions and to quantify co-localizations indicative of cell-cell interactions, we used the spatial image analysis of tissues (SPIAT) R package on the cell coordinates of cells in each cluster. The visualization of the spatial distribution of immune cells throughout the colon is exemplified in Fig. 1f, g. Next, the pairwise distances between the cells within one immune cell populations, or between two immune cell populations (clusters), were calculated using all cell-cell comparisons (range: 0 – 10,000 μm) at a time for all vehicle- and AOM/DSS-treated colons. The results are illustrated as violin plots, where a broad base indicates a higher number of cells in proximity. Our data show that at homeostasis (in the healthy, vehicle-treated colon), the average cell-to-cell distances varied between the different immune cell populations (Supplementary Fig. 3a), and the distances between most immune cells was reduced during colitis (following AOM/DSS treatment, Supplementary Fig. 3b). For example, the majority of neutrophils (cluster 4) were located between 2000 and 3000 μm of each other in vehicle-treated samples but were closer to each other (at distances of <1000 μm) in AOM/DSS-treated samples. As immune cells can vary in size from around 7–15 μm (B, T, and NK cells) to 15–30 μm (macrophages), a distance of 7–30 μm between two cell nuclei may indicate direct cell-to-cell interactions. To identify co-localizations indicative of cell-to-cell interactions, violin plots for distances measured between the center of two cells’ nuclei for all cell-cell comparisons within the range 0–20 μm were generated (Supplementary Fig. 3c, d, an * in the violin plot indicates *P* < 0.05 between vehicle- and AOM/DSS-treated samples). The number of comparisons for each pair of clusters (within the given distance) is given in Supplementary Data 1. In the inflamed colon, all immune cell types showed neighboring interactions within and between each population at distances below 20 μm (red line in figures), validating the applied cut-off. Notably, in the healthy colon (vehicle-treated samples/at homeostasis), close interactions (<20 μm) were noted primarily within each population, whereas few close interactions were identified between different immune cell populations (Supplementary Fig. 3c and Fig. 2a–c). For example, neutrophils (cluster 4) did not display close contact with any other immune cell types, except rare (*n* = 6) close interactions with M2-like macrophages (cluster 7, Supplementary Fig. 3c). During colitis, however, multiple interactions became more abundant, exemplified by interactions between Treg cells and DCs (Fig. 2a, from *n* = 13 to 143 comparisons), B cells and DCs (Fig. 2b, *n* = 15 to 113), and Treg cells and neutrophils (Fig. 2c, *n* = 1 to *n* = 2893, *P* = 0.014), as illustrated in corresponding images (Fig. 2d–f). Neutrophils now also displayed close interactions with Tc cells (*n* = 322) and DCs (*n* = 243, Supplementary Fig. 3d). These interactions represent known mechanisms of immune cell activity, including the binding between cellular receptors and transfer of antigens, supporting the accuracy of the analysis. A different trend was noted for M2-like macrophages and DCs, where close interactions were more common at homeostasis (*n* = 46) than during colitis (*n* = 26, Supplementary Fig. 3). In conclusion, using spatial analysis, we can identify and characterize the distribution and complex cell-cell interactions both in the healthy and inflamed colon and quantify close interactions between multiple immune cell types.

### Sex differences in the spatial organization of immune cell interactions

To investigate whether sex differences exist in the co-localizations of and interactions between immune cell populations, samples were stratified by sex and treatment (vehicle or AOM/DSS) and SPIAT analysis performed. Violin plots showing the pairwise distances between all cell-cell comparisons (range: 0 to 10,000 μm) are given in Supplementary Fig. 4. In vehicle-treated samples, differences were seen in the overall distribution of cell-to-cell distances between males and females. For example, the majority of Tc cells (cluster 5) and B cells (cluster 8) were located between 3000 – 5000 μm of each other in males, while they were closer (2000 – 3000 μm) in females (Supplementary Fig. 4). For cell-cell comparisons within the range 0 – 20 μm (Supplementary Fig. 5, a * in the violin plot indicates *p* < 0.05 between vehicle- and AOM/DSS-treated samples, the number of comparisons for each pair of clusters is given in Supplementary Data 2), significant sex differences were observed in both the healthy and inflamed colon. In the healthy (vehicle-treated) colon, close interactions between neutrophils (cluster 4) and M2-like macrophages (cluster 7) were specific for females (*n* = 6, with males displaying 0 close interactions (Fig. 2g, *P* = 0.047, Mann–Whitney test). Females also displayed slightly more close interactions between M2-like macrophages (cluster 7) and B cells (cluster 8) compared to males (Fig. 2h). Moreover, fewer neutrophils (cluster 4) were located in proximity to each other in males (*n* = 2) compared to females (*n* = 10) (Supplementary Fig. 5). Males, on the other hand, displayed a rare trend of interactions between Tc cells (cluster 5) and M2-like macrophages (cluster 7, *n* = 4 in males and *n* = 2 in females). In AOM/DSS-treated samples, we also observed differences in the overall distribution of cell-to-cell distances between males and females, with the majority of B cells (cluster 8) located closer (within 1500 μm) of each other in females compared to in males (within 2500–4000 μm, Supplementary Fig. 4c, d). Similarly, females displayed more DCs (cluster 3, *n* = 500 in females vs. *n* = 110 in males) and Tc cells (cluster 5, *n* = 124 in females vs. *n* = 70 in males) in proximity to each other (Supplementary Fig. 5c, d). Moreover, as in the homeostatic colon, more close interactions between M2-like macrophages (cluster 7) and B cells (cluster 8) were seen in females (*n* = 41) compared to males (*n* = 3) (Fig. 2i). The results of this analysis thus demonstrate apparent sex differences in immune cell distributions and colocalizations in the healthy and inflamed colon.

### Manual annotation confirms the colitis-induced immune cell landscape

**Fig. 3:**
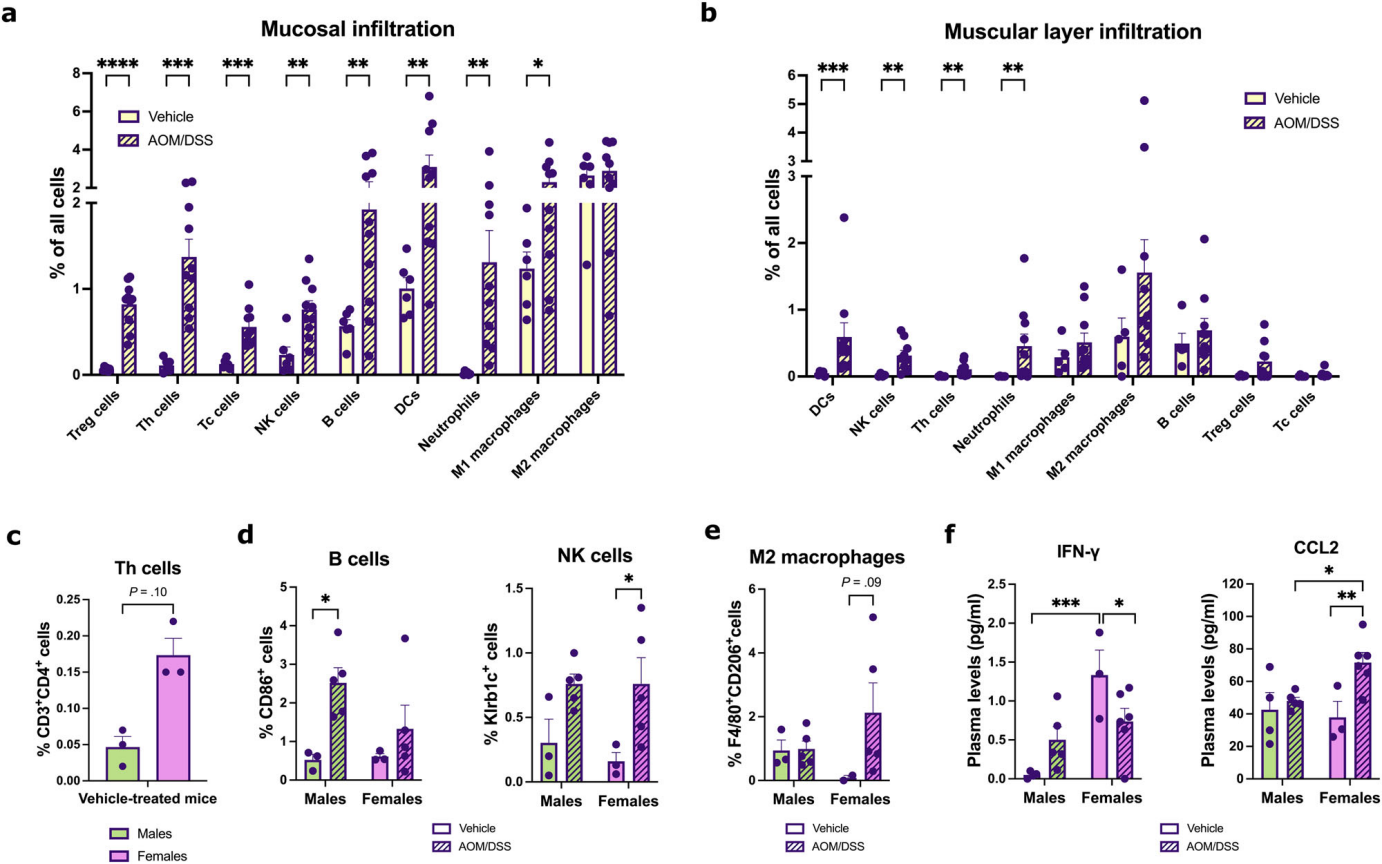
Quantification of immune cell infiltration in the healthy and inflamed colon and corresponding sex differences. Immune cell infiltration in the colonic (**a**) mucosa and (**b**) muscular layer in response to AOM/DSS treatment (vehicle: n = 6, AOM/DSS: *n* = 10 mice). Sex-stratified immune cell infiltration of (**c**) Th cells in the homeostatic mucosa (vehicle-treated, *n* = 3 per sex), (**d**) B and NK cells in the homeostatic and inflamed mucosa (vehicle: *n* = 3 per sex, AOM/DSS: *n* = 5 per sex), and (**e**) M2-like macrophage in the homeostatic and inflamed muscular layer (vehicle: *n* = 2-3 per sex, AOM/DSS: *n* = 5 per sex). **f** Sex-stratified plasma levels of IFNγ and CCL2 (vehicle: *n* = 4 males *n* = 3 females, AOM/DSS: *n* = 5 males and *n* = 6 females). Results are given as mean ± SEM. Statistical analysis was performed using multiple unpaired *t*-tests (**a**), multiple Mann–Whitney tests (**b**), Mann–Whitney test (**c**), and two-way ANOVA tests with Fisher’s LSD test (**d**–**f**). Males (green) and females (purple). *P < 0.05, **P < 0.01, ***P < 0.001, and ****P < 0.0001.

As unsupervised clustering did not clearly identify certain immune cell populations (including NK cells and M1-like macrophages) and we noted the above-mentioned limitations (regarding clusters 6 and 9), we also manually quantified the immune cell infiltration according to the markers in Table 1. For quantification of B cells, we excluded cells displaying CD86^+^F4/80^+^ (M1-like macrophages) and CD86^+^CD11c^+^ (DC) staining as well as triple-positive cells (CD86^+^F4/80^+^CD11c^+^ cells) that could constitute subtypes of these populations from the CD86^+^ cell population. The mucosa was further quantified separately from the muscular layer and the positive cells were counted (QuPath) in all annotated areas of all samples. Quantifications (percent of all cells) demonstrated that mucosal infiltration of all immune cell populations studied, except for M2-like macrophages, increased significantly during colitis (Fig. 3a). We also observed significant increases of colonic muscular layer infiltration during colitis of DCs, NK cells, Th cells, and neutrophils (Fig. 3b). When stratified by sex, females exhibited a trend (*P* = .10) of higher numbers of Th cells infiltrating the mucosa of the healthy colon (vehicle-treated) compared to males (Fig. 3c and Supplemental Fig. 7a, b). During colitis, only males displayed significantly increased B cell infiltration, while NK cell infiltration increased significantly only in females (Fig. 3d). Sex differences were also indicated in M2-like macrophage infiltration in the muscular layer. Females, which exhibited a minimal infiltration in the healthy colon, showed a trend of sex-specific increase upon colitis (*P* = 0.09, Fig. 3e). Notably, sex differences were corroborated by measurement of plasma cytokines. A multiplex assay for a panel of six cytokines was performed on plasma samples from mice in the same cohort (n = 17). On average, mice (sexes combined) exhibited significantly increased plasma levels of IL-6 (IL6), TNF-α, IL-17, CCL2, and IL-1β (IL1B), but not of IFNγ following AOM/DSS treatment (Supplementary Fig. 7c). However, when separated by sex, we noted that females displayed significantly higher levels of IFNγ at homeostasis and a decrease following treatment (Fig. 3f). Females also exhibited sex-specific increases in CCL2 (Fig. 3f), TNF-α, and IL-6 (Supplementary Fig. 7d) levels in response to treatment. In conclusion, sex differences in the infiltration of multiple immune cell populations were observed, quantified, and reflected in plasma cytokine profiles.

### Longitudinal spatial characterization of colonic immune cell infiltration

**Fig. 4:**
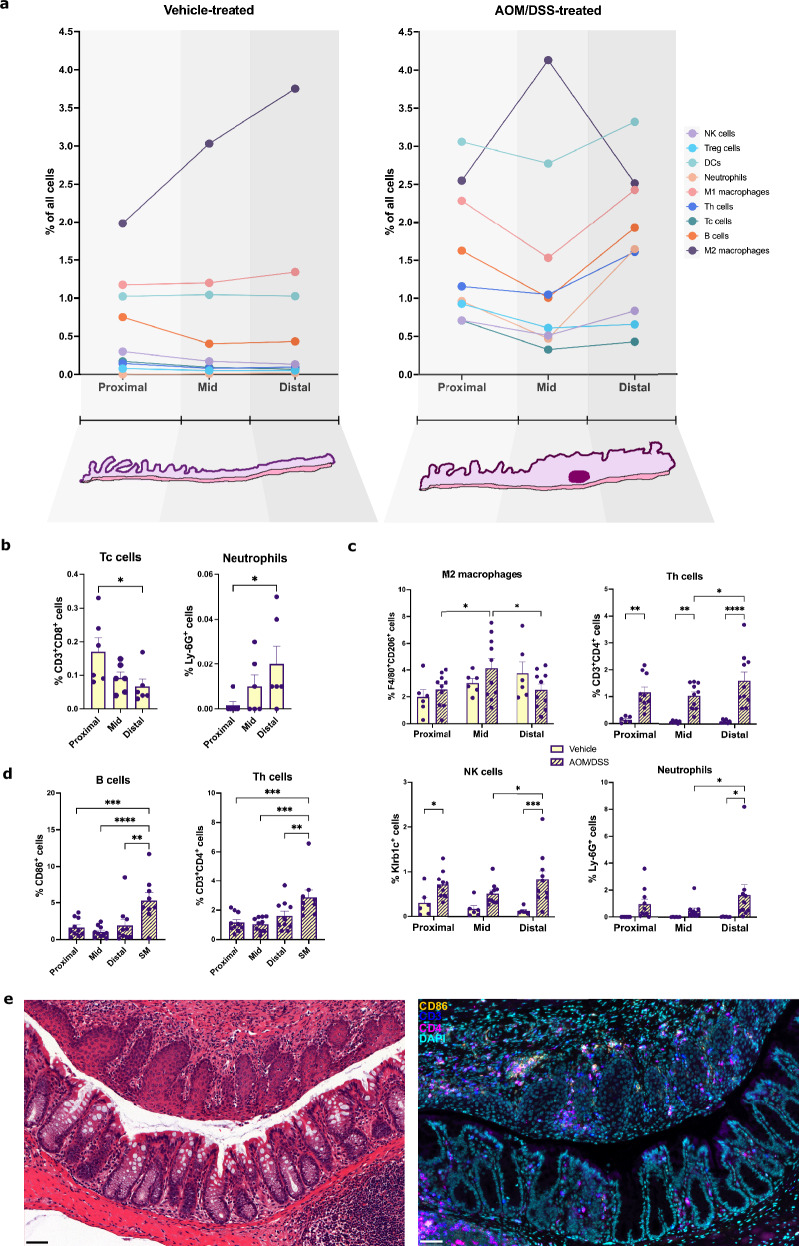
Mucosal immune cell infiltration displays distinct longitudinal differences at homeostasis and in response to colitis. **a** Graphs visualizing the longitudinal distribution of infiltrating immune cells in the colon at homeostasis (left, vehicle-treated, *n* = 6) and during colitis (right, AOM/DSS-treated *n* = 10 mice). **b** Tc cell and neutrophil infiltration in the different regions of the colon at homeostasis (n = 6 vehicle-treated mice). **c** Differences in M2-like macrophage, Th cell, NK cell, and neutrophil infiltration over the proximal-distal axis in response to colitis (*n* = 6 vehicle- and *n* = 10 AOM/DSS-treated mice). **d** B cell and Th cell infiltration in regions of squamous metaplasia compared to in different regions of the inflamed colon (*n* = 9-10 AOM/DSS-treated mice). **e** Representative images of a region of squamous metaplasia (top part of the image) compared to colonic epithelium (bottom part of the image). Left: Image of H&E-stained section. Right: Image of multiplex immunofluorescence for CD86 (orange), CD3 (blue), and CD4 (magenta). Results are given as mean ± SEM (**b**–**d**). Statistical analysis was performed using one-way ANOVA tests with Fisher’s LSD test (**b**, **d**) and two-way ANOVA tests with Fisher’s LSD test (**c**). *P < 0.05, **P < 0.01, ***P < 0.001, and ****P < 0.0001. Scale bars: 50 μm (**e**).

Next, we investigate differences in immune cell infiltration along the proximal-distal axis of the colon. The Swiss-rolled colon samples were divided into the proximal, mid, and distal colon, and the percentage of immune cells infiltrating the mucosa was calculated in each region. Large lymphoid follicles (Supplementary Fig. 6a, b) and distal regions of squamous metaplasia characterized by strong inflammation and immune cell infiltration (H&E-staining in Supplementary Fig. 6c) were noted specifically in AOM/DSS-treated mice and analyzed as separate entities. The spatial distribution of infiltrating immune cells throughout the colon in vehicle- and AOM/DSS-treated mice is visualized in line graphs (Fig. 4a and Supplementary Fig. 8a–c). Overall, the longitudinal distribution was relatively uniform in vehicle-treated mice, but M2-like macrophages and neutrophils exhibited a gradually increasing distribution and Tc cells a decreasing distribution along the proximal-distal axis (Fig. 4a, b and Supplementary Fig. 8d). Upon the induction of colitis, the abundance of all immune cell populations increased more in the proximal and distal colon than in the mid colon, except M2-like macrophages, which increased significantly in the mid colon (Fig. 4a and c, and Supplementary Fig. 8e). The regions of squamous metaplasia exhibited significantly higher infiltration of B, Th, NK, and Treg cells and neutrophils than any region of the colon, whereas M2-like macrophage and Tc cell infiltration were significantly lower compared to the mid- and proximal colon, respectively (Fig. 4d, e and Supplementary Fig. 9a). In conclusion, our results show that immune cell infiltration displays significant longitudinal differences both at homeostasis and during colitis. The infiltration of multiple populations, but not Tc cells and M2-like macrophages, displayed the greatest increases in the distal colon and in regions of squamous metaplasia following the induction of colitis.

### Sex differences in immune infiltration along the proximal-distal axis

**Fig. 5:**
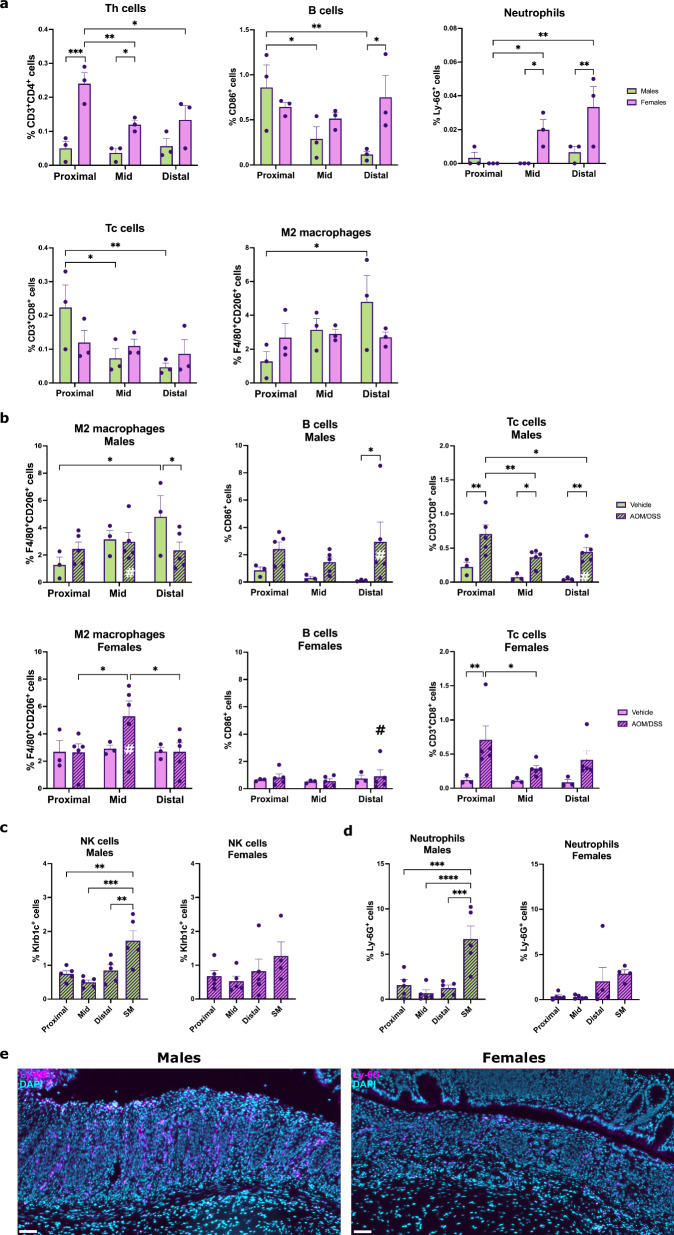
Sex differences in mucosal immune cell infiltration into different regions of the colon. **a** Th cell, B cell, neutrophil, Tc cell, and M2-like macrophage infiltration in the different regions of the colon at homeostasis (vehicle-treated, n = 3 for each sex). **b** M2-like macrophage, B cell, and Tc cell infiltration into the different regions of the colon in response to colitis (vehicle: *n* = 3 of each sex, AOM/DSS: *n* = 5 of each sex). **c** NK cell infiltration and (**d**) neutrophil infiltration in regions of squamous metaplasia compared to in different regions of the inflamed colon in treated males (*n* = 5) and females (AOM/DSS: *n* = 5 for regions of the colon and *n* = 4 for squamous metaplasia, per sex). **e** Representative images of neutrophil infiltration (Ly-6G in magenta) in regions of squamous metaplasia in males (left) and females (left). Results are given as mean ± SEM (**a**–**d**). Statistical analysis was performed using two-way ANOVA tests with Fisher’s LSD test (**a**, **b**) or one-way ANOVA tests with Fisher’s LSD test (**c**, **d**). *P < 0.05, **P < 0.01, ***P < 0.001, and ****P < 0.0001. # indicates significant sex differences (**b**). SM = squamous metaplasia (**c**, **d**). Scale bars: 50 μm (**e**). Males (green) and females (purple).

To explore sex differences in immune cell infiltration along the proximal-distal axis of the colon, the cell quantification above was stratified according to sex. This revealed that the higher levels of Th cell infiltration noted in females at homeostasis (vehicle-treated) was present along the longitudinal length of the colon but was pronounced (*p* < 0.001) in the proximal colon (Fig. 5a). While females exhibited a gradient along the proximal-distal axis, males, in contrast, had more evenly distributed (and lower) levels throughout the colon (Fig. 5a). Females further exhibited significantly higher levels of B cells in the distal colon and of neutrophils in the mid and distal colon and compared to males (Fig. 5a). Males, on the other hand, exhibited a gradient with higher numbers of B and Tc cells in the proximal colon compared to the mid and distal colon and an increase in M2-like macrophage infiltration along the length of the colon not seen in females. (Fig. 5a). No significant sex or regional differences were seen in the infiltration of M1-like macrophages or Treg, NK, or DC cells in males and females at homeostasis (Supplementary Fig. 9d). During colitis, the higher B cell infiltration noted above in males could be observed along the length of the colon and was pronounced in the distal colon (Fig. 5b). Further, Tc and Th cell infiltration increased significantly in each region of the colon in males, whereas infiltration was less pronounced in females (Fig. 5b and Supplemental Fig. 9e). Sex differences in longitudinal distribution were also noted during colitis. Higher numbers in the proximal versus distal colon were noted for Treg cells in males only (Supplementary Fig. 9e), whereas the increase in M2-like macrophage infiltration in the mid colon was specific for females (Fig. 5b). Additionally, the higher infiltration of NK cells and neutrophils in regions of squamous metaplasia was pronounced in males (Fig. 5c-e), while corresponding DC cell infiltration was pronounced in females (Supplementary Fig. 9b, c). Overall, multiple longitudinal sex differences in immune cell infiltration were apparent at both homeostasis and in response to treatment-induced colitis.

## Discussion

A general spatial characterization of the colonic immune microenvironment has been lacking. In this study, we used the novel COMET platform for multiplex immunofluorescence to profile the spatial immune landscape of the murine colon in both sexes, visualizing and characterizing co-localizations and interactions between infiltrating immune cell populations during colitis. Unsupervised clustering resulted in clusters corresponding to the expected identities for five immune cell populations (Tregs, DCs, neutrophils, Tc cells, and M2-like macrophages). Spatial analysis of the generated clusters enabled us to quantify interactions between multiple immune cell populations simultaneously as well as how these changed upon the induction of colitis. We identified cell-cell co-localizations indicative of direct interactions between, for example, Treg cells and DCs, DCs and B cells, and neutrophils and DCs. These cell types are known to communicate directly via cell-cell contact, direct transfer of antigens, and direct interactions via cellular receptors, respectively^47–49^, and their identification validates the accuracy of the analysis. Our analysis also indicated interactions between neutrophils and Tc cells. While these cell types communicate mainly indirectly via cytokines, antigen presentation by neutrophils has been reported and direct interactions are possible^50^, and this is supported by our data. By performing SPIAT analysis on sex-stratified samples, we profiled sex differences in the co-localizations of and interactions between immune cell populations and found that the close interactions between neutrophils and M2-like macrophages in the healthy colon were specific for females. Neutrophils and macrophages work together to promote the immune response against pathogens^51^, and our observation could be linked to the reported stronger immune responses seen in females^31^. Interestingly, while the cell-cell interactions (<20 μm) between different immune populations were rare in the normal (vehicle-treated) colon, close interactions were more common within the populations. The bases of these violin plots were especially broad, indicating a higher likelihood for very close interactions (5–8 μm) compared to slightly more distant interactions (10-20 μm). This differs from the pattern of interactions between different populations and also from the pattern following treatment and was especially clear for DCs, M2-like macrophages, and B cells and, in males only, for Tc cells. An interpretation of this is that the noted pattern could be indicative of cell divisions. While immune cells primarily proliferate in specific niches (for example, B cells proliferate in the spleen and other peripheral lymphoid tissues) and migrate to distant tissues such as the colon, tissue-resident macrophages, DCs, and T cells have been reported to self-renew^52–54^. Our observations could support the indication that several immune cell populations proliferate in the colon at homeostasis, but may also reflect other interactions or activities, such as between DCs, that maintain immune tolerance.

Unsupervised clustering analysis did not result in clear clusters corresponding to B cells, Th cells, M1-like macrophages, or NK cells. Instead, the cluster that contained CD86^+^ cells (cluster 8), presumed to be primarily B cells, also contained other cells positive for CD11c or Ly-6G, and cluster 9 included cells positive for multiple markers. CD86, the B cell marker used in this study, is expressed in B cells and upregulated upon B cell activation (B cells express CD86 on average at 10 TPM, per RNA-seq). However, DCs and macrophages^55^ can express trace levels of this marker (e.g., macrophages around 1 TPM^56^). Therefore, we quantified the CD86^+^ B cell population by excluding cells that co-expressed macrophage and DC markers (CD86^+^F4/80^+^, CD86^+^CD11c^+^, and CD86^+^F4/80^+^CD11c^+^ cells). The remaining CD86^+^ population, as analyzed here, is likely enriched for and representative of activated B cells. We cannot, however, exclude that it contains other cell types. We attempted to include the B cell-specific marker CD19 but were unable to obtain sufficiently specific staining for our conditions and setup, which is a limitation of our analysis.

Overall, manual quantification of immune cell infiltration enabled the classification of cell populations based on established markers and the use of appropriate thresholds for staining intensity for all markers in all samples. In addition to validating several of the results generated by unsupervised clustering and SPIAT analysis, this also provided a high-quality analysis due to the rigorous review of the staining in each channel separately. Our findings of increased infiltration of B, DC, Th, Tc, and Th cells and neutrophils during colitis (Fig. 3a) align with earlier findings, including studies that have used single-cell RNA sequencing to show that the proportions of cells in clusters containing B cells, plasmacytoid DCs, and T cells increased^57^, flow cytometry and immunohistochemistry to show that numbers of helper, cytotoxic, and regulatory T cells, macrophages, and B cells in the colon increased^58^, and a recent study utilizing single-cell RNA sequencing and flow cytometry to demonstrate that there were few neutrophils in the colonic lamina propria at steady state (homeostasis) but that their number significantly increased during DSS-induced colitis^59^.

The M1/M2 paradigm of macrophage polarization is a simplification that is debated and not entirely representative of the complexity of macrophage activation^60^. We, therefore, refer to these populations as M1-like and M2-like macrophages to indicate the likelihood that these macrophages are more pro-inflammatory or anti-inflammatory. A recent study used spatial transcriptomics at single-cell resolution to profile macrophages and neutrophils in the intestine of healthy controls and IBD patients. The authors identified several subtypes of macrophages and found that both M0 and M2 macrophages were present in the healthy colon. Samples from IBD patients additionally contained inflammatory/activated M1 macrophages in several different states, although these macrophages did not display the full characteristic M1 signature, further confirming the diversity of macrophage phenotypes^61^. Further, single-cell analysis of Crohn’s disease lesions identified gut-resident macrophages lacking pro-inflammatory genes and expressing CD206, as well as inflammatory macrophages with increased expression of NF-κB. The frequencies of inflammatory macrophages were increased, and the frequencies of resident macrophages decreased, in inflamed samples compared to uninflamed samples from the same patients^28^. Single-cell profiling of a cohort of pediatric IBD patients also found that the infiltration of hyper-inflammatory macrophages and DCs was a hallmark of pediatric IBD^27^, and another group found that a population of inflammatory IL-1β^+^ macrophages/monocytes was expanded in the mucosa of patients with active Crohn’s disease and ulcerative colitis^29^. Therefore, several aspects of the described immune landscape of the mouse colon in our study is validated by studies in the human colon.

Here, we report that immune cell infiltration in response to AOM/DSS-induced colitis displayed distinct regional differences along the proximal-distal axis of the colon. The strongest response (including in Th cell, NK cell, neutrophil, B cell, and DC infiltration) occurred in the distal colon. This is in line with findings that show that the distal colon is the region most affected during colitis^43^ and that the tumors arising in the AOM/DSS model primarily develop in this region^32,62^. In the homeostatic colon, the highest numbers of B cells (Supplementary Fig. 8d) and Tc cells (Fig. 4b) were seen in the proximal colon. This is consistent with findings from a study investigating regional colonic immune cell infiltration in healthy volunteers^63^. Memory B cells have also been found to predominate in the proximal colon^64^. However, a study in healthy human donors found that B cells are more abundant in the sigmoid colon compared to the proximal colon^65^. A study by Frede et al. showed that elevated B cell numbers during intestinal inflammation were mainly seen in tertiary lymphoid structures, which appeared to be located mainly in the distal colon^17^. While we did not quantify immune cell infiltration in these structures (due to the density of cells not allowing accurate cell segmentation), we also observed the greatest numbers of B cells in the distal colon during colitis (Supplementary Fig. 8e), supporting the notion that B cell infiltration during intestinal inflammation is prominent in the distal colon. Additionally, we demonstrated significantly higher infiltration of B, Th, NK, Treg, and neutrophils in regions of squamous metaplasia compared to all regions of the colon. Squamous metaplasia has been documented in patients with ulcerative colitis-associated cancer and is indicative of repeated wound-repair cycles in response to chronic inflammation^66^, which may explain the augmented immune cell infiltration we observed. Overall, our study provides a spatial analysis of immune infiltration during colitis that contributes to the understanding of the complex interplay between different immune populations in the inflamed intestine.

Anti-inflammatory M2 macrophages can suppress colitis and are known to decrease in abundance during DSS-induced colitis^37^. The results of the unsupervised clustering analysis show a decrease in M2-like macrophage infiltration upon AOM/DSS treatment. Our manual longitudinal and sex-stratified quantification demonstrated that this was specific for the distal colon of males, whereas females exhibited a mid-colon increase. Sex differences in innate and adaptive immunity are well-documented in mammals. For example, women have an increased susceptibility to autoimmune diseases and men to non-reproductive malignant cancers. Females have higher CD4^+^ T cell counts than males, while males display higher NK cell frequencies^31^. Further, women have higher numbers of proliferating CD4^+^ T cells in the gut compared to men^67^. In line with this, in the homeostatic colon, we observed a trend of higher Th cell numbers in females (Fig. 3c) and higher NK cell numbers in males (Fig. 3d), validating the presence of the observed sex differences also in the murine colon. However, there are few studies that investigate differences in immunity as seen in immune cell infiltration of the colon. A study by Elderman et al. used FACS to demonstrate more DCs, macrophages, and NK cells but fewer T cells in intestinal Peyer’s patches in male mice compared to female mice at baseline^68^. Although our study did not investigate the immune composition of Peyer’s patches, we also observed a trend of fewer Th cells in the colon of male mice at baseline (Fig. 3c). Our study took care to focus on sex differences in colonic immune cell infiltration, and we noted distinct differences in Th, Tc, and Treg cell infiltration. While these populations were often higher at homeostasis in females, males displayed greater increases in Th and Tc cell infiltration upon treatment. Th and Tc cells can have pathogenic roles during intestinal inflammation by promoting the secretion of pro-inflammatory cytokines^13,15,69^, but mucosal T cells also play important roles in maintaining the balance between immune activation and tolerance at mucosal sites^70^. The higher levels in the healthy female colon may be indicative of females’ increased risk of autoimmune inflammatory bowel disorders. Further, B cell infiltration exhibited one of the strongest sex differences. Activated B cells disrupt the epithelial-stromal crosstalk during intestinal regeneration and B cell expansion during intestinal injury impairs mucosal healing during colitis^17^. The pattern of B cell infiltration that we note here is thus indicative of a more serious phenotype in the distal colon in males. As this was accompanied by a decrease of the inflammation-antagonizing M2-like macrophages in the distal colon of males during colitis, this likely contributes to the noted sex difference of more severe colitis and increased tumor formation in males^23,44^. That males were more prone to inflammation was corroborated at the systemic level in our study, by a lower baseline IFNγ plasma levels and lower levels of CCL2 during colitis than females. IFNγ is a primary activator of macrophages and plays a critical role in the development of colitis, driving leukocyte infiltration and mucosal damage^71^, while CCL2 is a potent chemoattractant and activator of monocytes and macrophages^72^. The lower systemic levels of these cytokines in males may reflect the general sex differences in innate and adaptive immunity, especially the observed differences in M2-like macrophage infiltration. The slightly higher steady-state levels and greater increase of plasma IL-17 levels in males during colitis may be a consequence of the greater increase in colonic Th cell infiltration seen, since a subset of Th cells, Th17 cells, are a key source of IL-17^73^. Plasma levels of TNF-α, on the other hand, were significantly increased upon treatment only in females. Since TNF-α is produced in large quantities by NK cells (as shown in IBD patients^74^), this difference may be linked to the higher mucosal NK cell infiltration in females during colitis. We therefore conclude that sex differences in colonic immune cell infiltration are reflected in the systemic response of males and females, which may have clinical implications. Anti-TNF therapy is used to reduce inflammation and promote mucosal healing in IBD patients, and a recent study of ulcerative colitis patients found that men were less likely than women to achieve clinical remission, mucosal healing, and clinical response during the induction phase of TNF inhibitor treatment^75^. By specifying key differences in immune cell populations in the male and female colon and characterizing the pro-inflammatory, tumor-promoting immune microenvironment in the distal colon of males, our study provides a mechanistic explanation for the noted sex differences in colitis, IBD, CRC development, and the response to treatment. Our findings align with the increased risk of CRC observed in men^20^, especially in IBD patients^18^, compared to women. Our data thus characterize and explain some of the noted sex differences in innate and adaptive immunity, with a focus on the murine colon.

Strengths of our study include the simultaneous profiling of multiple immune cell populations with full spatial context and including their cell-cell interactions, enabled by the COMET platform, and performed both at homeostasis and during colitis in both sexes, which has not been previously performed. Another strength is the characterization of immune cell infiltration in regions of squamous metaplasia. A limitation of our study is that multiple subtypes and other immune cell populations that are present in the microenvironment were not investigated. This is due to the fact that we used stringent parameters for antibody testing and optimization, both manually and on the COMET platform. This excluded multiple markers as the antibodies did not pass our quality criteria for the assay. As a result, we are confident that the quality of our data is high, although additional studies are required for comprehensive subclassification of the immune cell populations. Further, while the unsupervised clustering analysis led to the identification of nine clusters and several clearly reflected specific cell populations, two clusters were less conclusive. We verified rare incidences of nonspecific staining that were collected within the smallest cluster (cluster 9), but the small number of cells in this cluster is unlikely to skew the overall results of the clustering analysis (or to interfere with the results of the quantification). Our identification of DC cells using the marker CD11c could be affected by the fact that other immune cell types can also express CD11c. However, RNA-seq data demonstrates that CD11c is expressed at considerably higher levels in DCs (601 TPM) compared to other cell types (34 TPM in B cells and 3 TPM in monocytes) in mice^76^ (data set was accessed on 2023-08-14 via the Expression Atlas^77^https://www.ebi.ac.uk/gxa/experiments/E-MTAB-3079/Results?geneQuery=%5B%7B%22value%22%3A%22cd11c%22%7D%5D), and thus, the approximation used here should be relatively accurate for DCs overall. Our analysis is unable to distinguish neutrophils and polymorphonuclear myeloid-derived suppressor cells (PMN-MDSCs) from each other, as Ly-6G marks both types of cells and there are currently no phenotypic cell surface markers that enable their separation in mice. PMN-MDSCs and neutrophils both have dual roles in IBD, with pro- and anti-inflammatory effects documented^78–80^. Moreover, the spatial analysis measuring the distances between cells is naturally affected by the physical locations imposed by the Swiss-rolled colons and larger distances can be artificially affected and not physiologically relevant. The number of cells quantified in the muscular layer was also fewer than in the mucosa due to the generally smaller area of this region and the presence of high background signal, leading to the exclusion of some areas. Therefore, the results of the quantification in the muscular layer have less power than those from the mucosa. Finally, it is to be noted that software-based image quantification is not perfect and that the results will inevitably vary slightly depending on the methods and classifiers used.

In conclusion, we have performed spatial profiling of the colonic immune microenvironment at homeostasis and during colitis in mice and have also investigated sex differences in immune infiltration. We characterize close interactions between immune cell populations in both the healthy and inflamed colon and reveal female-specific interactions between neutrophils and M2-like macrophages in the healthy colon. We show that the distal colon is the region most affected during colitis, with significantly increased infiltration of multiple pro-inflammatory immune cell populations and decreased infiltration of anti-inflammatory M2-like macrophages, especially in males, and we define the extensive immune infiltration into regions of squamous metaplasia. To the best of our knowledge, this is the first study to provide a broad spatial profiling of the colonic immune landscape at homeostasis and during colitis, including the identification of close interactions between immune cell populations and analysis along the proximal-distal axis and of sex differences. Our findings provide a basis for the understanding of male and female intestinal inflammatory diseases and potential new avenues to modulate the inflammatory immune microenvironment. This can also be relevant for studies of immune checkpoint inhibitors and their efficacy in colorectal cancer.

## Methods

### In vivo experiments

In this study, we used the AOM/DSS model of colitis-associated cancer. This model is based on an intraperitoneal injection of AOM to induce carcinogenesis, followed by three cycles of DSS in drinking water to induce inflammation, leading to colitis and rapid tumor development in the distal colon (within 15 weeks of initiation of treatment)^32^. The animal study and sample collection were previously performed and related data has been reported^23^. In short, 5 – 10 week-old mice of both sexes on a C57BL/6 J background (controls in our previous study^23^, *N* = 16 mice total) were randomly assigned to treatment with vehicle (0.9% NaCl, *n* = 6 mice) or AOM/DSS (*n* = 10 mice) and sacrificed 9 weeks after initiation of treatment. Mice were fed a standard soy diet, with food and water provided ad libitum. Mice were given one intraperitoneal injection with vehicle (saline) or 10 mg/kg body weight AOM (Sigma-Aldrich, St. Louis, MO, USA) on day one of the first week, after which the mice injected with AOM were given 2.5% DSS (MP Biomedicals, Santa Ana, CA, USA) in their drinking water ad libitum the second week, while vehicle-treated mice were provided with normal drinking water without DSS. All mice were given normal drinking water for 2 weeks following DSS administration, after which the DSS cycle was repeated twice for AOM-injected mice, for a total of 3 three-week cycles of DSS followed by normal drinking water. AOM/DSS-treated mice were assessed daily until recovery. Mice were sacrificed at 9 weeks after vehicle or AOM injection, or earlier if they developed rectal prolapse and/or lost >20% of their body weight. Mice sacrificed before the endpoint of the study were excluded. Mice were anesthetized with isoflurane prior to cervical dislocation and confirmation of euthanasia. The in vivo study and all experimental protocols were approved by Linköpings djurförsöksetiska nämnd (Dnr ID 211) and were carried out in compliance with all relevant ethical regulations for animal testing and research.

### Sample collection

Blood was collected by cardiac puncture at sacrifice using EDTA-treated tubes and plasma was collected after centrifugation and stored at -80 °C for further analysis. Colons were harvested, washed, opened longitudinally, rolled into Swiss rolls, and fixed in 4% paraformaldehyde for 24 h, then preserved in 70% EtOH and embedded in paraffin.

### Tissue preparation and antigen retrieval

Tissues were sectioned into 4 μm thick sections and mounted on microscope slides with placement in the middle of the slides so that they fit within an area of 9 × 9 mm. Slides were baked at 55 °C for 25 min. Antigen retrieval was done using a TintoRetriever pressure cooker (Bio SB from D.B.A. ITALIA s.r.l.) at 114–121 °C for 9 min at pH9 with antigen buffer (EDTA Decloaker, BioCare Medical, CB917L). Slides were stored in a multi-staining buffer (BU06) before staining.

### Multiplex immunofluorescence using the COMET platform

Multiplex immunofluorescence was performed by the Spatial Proteomics National Facility (SciLifeLab, Stockholm, Sweden) using the commercial COMET platform (Lunaphore Technologies, Tolochenaz, Switzerland). The COMET platform is an automated staining and imaging platform that provides high-throughput multiplex staining with full spatial context. COMET uses a patented microfluidic staining technology to perform rapid sequential immunofluorescence. Repeated cycles of staining, imaging, and elution are performed, and the acquired images are automatically stitched and stacked together by the instrument’s software. The COMET platform uses two antibodies per cycle, detected in TRITC and Cy5 channels, co-stained with the nuclear stain DAPI (4’,6-diamidino-2 phenylindole, dihydrochloride). All stainings were performed using commercially available buffers and preparation protocols from Lunaphore: Multistaining buffer (BU06), elution buffer (BU07), quenching buffer (BU08), imaging buffer (BU09) and blocking buffer (BU10). We used the COMET platform to stain colon tissue samples for a panel of 10 markers, given in Table 1.

Before antibody staining cycles commenced, blank images of the TRITC and Cy5 channels were captured for autofluorescence subtraction during post-acquisition image processing. A blocking step of a 1 min incubation with blocking buffer (BU10, 2,5% normal goat serum) preceded all staining cycles except cycle 3 (CD3 + FOXP3). Primary antibodies were diluted to the desired concentrations (see Table 1) in SignalStain Antibody Diluent (Cell Signaling Technology, #8112) and incubated for 8 min in each cycle. Secondary antibodies in the TRITC channel (Alexa Fluor goat anti-rat AF555+ (A48263)) were diluted to a concentration of 1:100 while Cy5 channel secondaries (Alexa Fluor 647 goat-anti-rabbit (A21245)) were diluted to 1:200, all in multistaining buffer. Secondary antibodies were incubated for 8 min for all but cycle 7 (F4/80), where it was 4 min, along with DAPI (PL220, Invitrogen), followed by image acquisition. Exposure time for DAPI was 100 ms and for TRITC and Cy5 exposure times were differently optimized (see Supplementary Data 3). DAPI images were acquired in every staining cycle to ensure alignment of cycles during image processing. All images were acquired with a CMOS IDS UI-3280CP-M-GL camera (2456 px x 2054 px, 0.23 μm per pixel) and a 20X objective 0.75 NA/1 mm WD (CFI P-Apo 20X Lambda/0.75/1.00). Following image acquisition, antibodies in every cycle were eluted for 4 min, except in cycle 7 (F4/80), where the elution time was 2 min, using Lunaphore elution buffer (BU07). All samples were prepared and stained in batches of two or four on the COMET platform.

All antibodies were selected, validated, and optimized by manual single-plex immunofluorescence prior to inclusion in the panel. Secondly, they were optimized on the COMET platform according to protocols for optimization and sequential IF provided in the COMET software before the final multiplex staining was performed. The order of antibodies in the panel was decided based on epitope stability and elution efficiency, with stable epitopes and hard-to-elute antibodies in the later cycles. The antibodies included in the final panel and their dilutions are given in Table 1. A total of 16 formalin-fixed, paraffin-embedded Swiss-rolled colons samples from mice of both sexes that had undergone 9 weeks of treatment with vehicle or AOM/DSS were analyzed. The parameters for the multiplex staining are provided in Supplementary Data 2.

### Immune cell population clustering and mapping

Clustering was performed on the per-cell-intensities of all markers in all cells quantified from all samples. Treg cells from one specific sample constituted the vast majority of all cells in that cluster. Upon manual verification, these cells were not overrepresented in this sample, but the CD3 and FOXP3 staining in this particular sample was weak and displayed high background. Despite normalizing data from this sample, this led to skewing of the results of the clustering analysis, and this sample was therefore excluded. The per-cell-intensities of all markers were extracted from QuPath and used for unsupervised clustering analysis in R (v. 4.3.0)^81^. For that, all intensities were normalized with range normalization (caret, v. 6.0-94) transforming the values between 0 and 1 to account for differences between samples and merged into one file. FlowCore (v.2.12.0)^82^ was used to transform the data to a flowFrame object and FlowSOM (v.2.8.0)^83^ was used for unsupervised clustering. The number of clusters was chosen manually according to the expected staining results. The resulting clusters were visualized using heatmap (pheatmap, v.1.0.12)^84^. tSNE (Rtsne, v.0.16)^85^ was used for mapping. Firstly, all cells were mapped with colors determined by the clusters. Then, to visualize immune cell populations only, cells that were negative for all markers (non-immune cells) were removed, and the remaining subset was re-mapped. All results except the heatmap were visualized using ggplot2 package (v.3.4.2)^86^.

### Spatial analysis using SPIAT

To visualize the spatial distribution of immune cell populations and investigate possible co-localizations, pairwise distance analysis was performed using the SPIAT (v.1.2.3)^87^ package in R. The samples were processed separately, applying cluster information for each cell per sample, and taking centroid coordinates for each cell. Distances were measured between the centers of each nucleus. Clusters 2 – 8 were deemed to be clusters of interest, and pairwise distances were calculated between the centroids of each cell belonging to either the same cluster (to explore cell-cell clustering) or two different clusters (to visualize distances between cells of two different populations). For example, if the distances between 1000 cells in a cluster were compared to each other, the distances were first compared between cell 1 and the other 999 cells, then between cell 2 and all remaining cells, and so on, for all cells in the cluster. Hence, the number of comparisons can be much greater than the number of cells in a cluster. The results were then merged for all samples and plotted using violin plots. The plots were plotted as full plots showing all distances (range 0 to 10 000 μm), plots showing the range 0–50 μm (for distribution overview), and plots showing the range 0–20 μm (where 20 μm was deemed the cutoff for the probable interaction limit). Regarding cell-cell interactions/colocalizations, the shortest cell-to-cell distances from nucleus-to-nucleus (5–20 um depending on cell type), indicate that two cells are next to each other. Direct side-to-side location was validated by manual inspection of images. As demonstrated in our analysis, such proximity was not random, and certain cell types have a statistically significant likelihood of physical interaction/closeness. While this does not per se demonstrate that they actively interact through, for example, receptor binding, several cell types that are known to actively interact were found in this analysis. We, therefore, consider our data to support the likelihood of direct (albeit not necessarily active) interactions. Lastly, the location of all cells in cluster 1–9 was plotted and overlaid onto the sample (colon roll) using the same package.

### Quantification of multiplex staining

For quantification of the multiplex staining performed on the COMET platform, images were analyzed using the open-source software QuPath^88^. Each sample was annotated manually, with the mucosa (including intestinal epithelial cells) and the muscular layer being analyzed separately. Areas with edge effect, dust, or nonspecific staining were excluded. Cells were detected using the StarDist extension, a deep-learning-based method for nucleus detection^89^. Single-measurement and composite classifiers were subsequently created for each staining/channel individually. For each classifier, “Channel filter” was set to the relevant channel (e.g., “CD3”), “Measurement” was set to the relevant parameter (e.g., “Cytoplasm: Mean”, see Table 1 for the settings used), and “Threshold” was adjusted to obtain accurate quantification of positive cells. We thoroughly evaluated which classifier settings (“Measurement”) were best for each marker. FOXP3 translocates to the nucleus in Treg cells^90–92^, and consistent with this, the FOXP3 staining we observed always overlapped with the nucleus (Fig. 2d and f). For FOXP3, we therefore used the specified classifier setting (“Nucleus: Mean”). Ly-6G is located in the cell membrane, but the staining partially obscured the nucleus, and the use of the classifier setting “Nucleus: Max” resulted in the most accurate quantification of positive cells (as exemplified in Supplementary Fig. 10) and is recommended in such cases^93^. For CD8 and CD11c, we used the classifier setting “Cell: Mean” rather than “Cytoplasm: Mean”, as this provided the most accurate quantification of positive cells (as exemplified in Supplementary Fig. 11). M1-like macrophages were defined as F4/80^+^CD86^+^ cells and M2-like macrophages as F4/80^+^CD206^+^ cells. The numbers of CD86^+^ cells were quantified by excluding CD86^+^F4/80^+^ cells, CD86^+^CD11c^+^ cells, and CD86^+^F4/80^+^CD11c^+^ cells, enabling the quantification of a population likely enriched in activated B cells. The classifiers were run one by one for all annotations in one sample, and the results were recorded. Thresholds for the classifiers were manually adjusted between samples due to varying staining intensity. The percentage of positive cells was calculated from the sum of all cells and positive cells from each sample. The colon rolls were divided into three areas of approximately equal length; the proximal colon, mid colon, and distal colon, and mucosal immune cell infiltration was also quantified separately for each region. Quantification was performed blinded to sample genotype and sex.

### Plasma cytokine assay

Plasma cytokine assay was performed by the Affinity Proteomics Infrastructure Unit (SciLifeLab, Stockholm, Sweden) using Simple Plex assays run on the Ella Automated Immunoassay System (ProteinSimple, R&D Systems, Bio-Techne) according to the manufacturer’s instructions. A total of 18 plasma samples from mice of both sexes that had undergone 9 weeks of treatment with vehicle or AOM/DSS were analyzed. Samples were run in triplicate and sample concentration values provided in pg/mL by fitting relative fluorescence units to calibration curve parameters. The procedure has been described in detail by Aldo et al.^94^. Plasma levels of IFNγ, IL-6, TNF-α, IL-1β, IL-17, and CCL2 were analyzed.

### Statistics and reproducibility

Statistical analysis was performed in GraphPad Prism 9 (GraphPad Software, San Diego, CA, USA) using unpaired *t*-tests, Mann–Whitney tests, one-way ANOVA tests with Fisher’s LSD test, or two-way ANOVA tests with Fisher’s LSD test as described in the figure legends. Normality was tested using Shapiro–Wilk tests. The results are presented as mean ± SEM. A *P*-value of ≤0.05 was considered statistically significant (*P ≤ 0.05, **p ≤ 0.01, ***p ≤ 0.001, ****p ≤ 0.0001). Sample size was estimated prior to the implementation of the experiment to ensure statistical power of detection. Sample size is reported as a number where relevant. The antibody panel used for the multiplex staining was thoroughly optimized and all antibodies included in the final panel were rigorously tested for specific staining and sufficient staining intensity to ensure reproducibility.

### Reporting summary

Further information on research design is available in the Nature Portfolio Reporting Summary linked to this article.

## Supplementary information

Supplementary Information

Description of Additional Supplementary Files

Supplementary Data 1

Supplementary Data 2

Supplementary Data 3

Supplementary Data 4

Reporting summary

## Data Availability

Numerical source data for graphs and plots in the main figures can be found in Supplementary Data 4. Additional data supporting the findings of this manuscript are available upon request.

## Code availability

The Spatial Image Analysis of Tissues (SPIAT) R package used in this study is publicly available online at https://trigosteam.github.io/SPIAT/. The modified scripts used for clustering, dimensionality reduction, and spatial analysis are available at GitHub (https://github.com/Linastep-bot/spatial-profiling-of-colonic-immune-microenvironment) and have also been deposited in Zenodo^95^.
