# Supplementary material for: Spatial profiling of the mouse colonic immune landscape associated with colitis and sex
Source: Commun Biol. 2024 Nov 29;7:1595. doi: 10.1038/s42003-024-07276-1 (PMC11606951; doi:10.1038/s42003-024-07276-1)
Supplement: Supplementary file 1 — Supplementary Information [file 42003_2024_7276_MOESM1_ESM.pdf]

Supplementary Figure 1

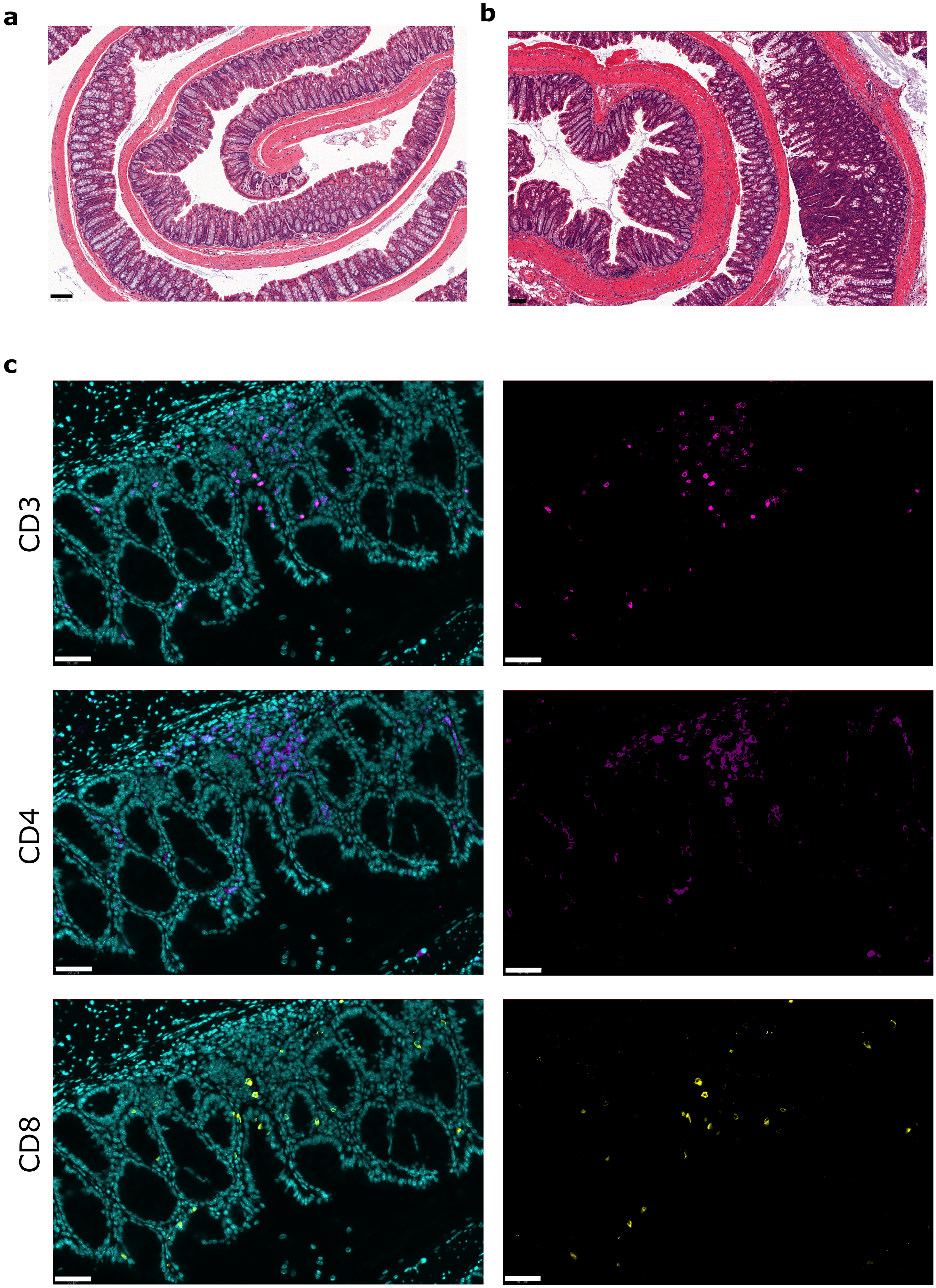

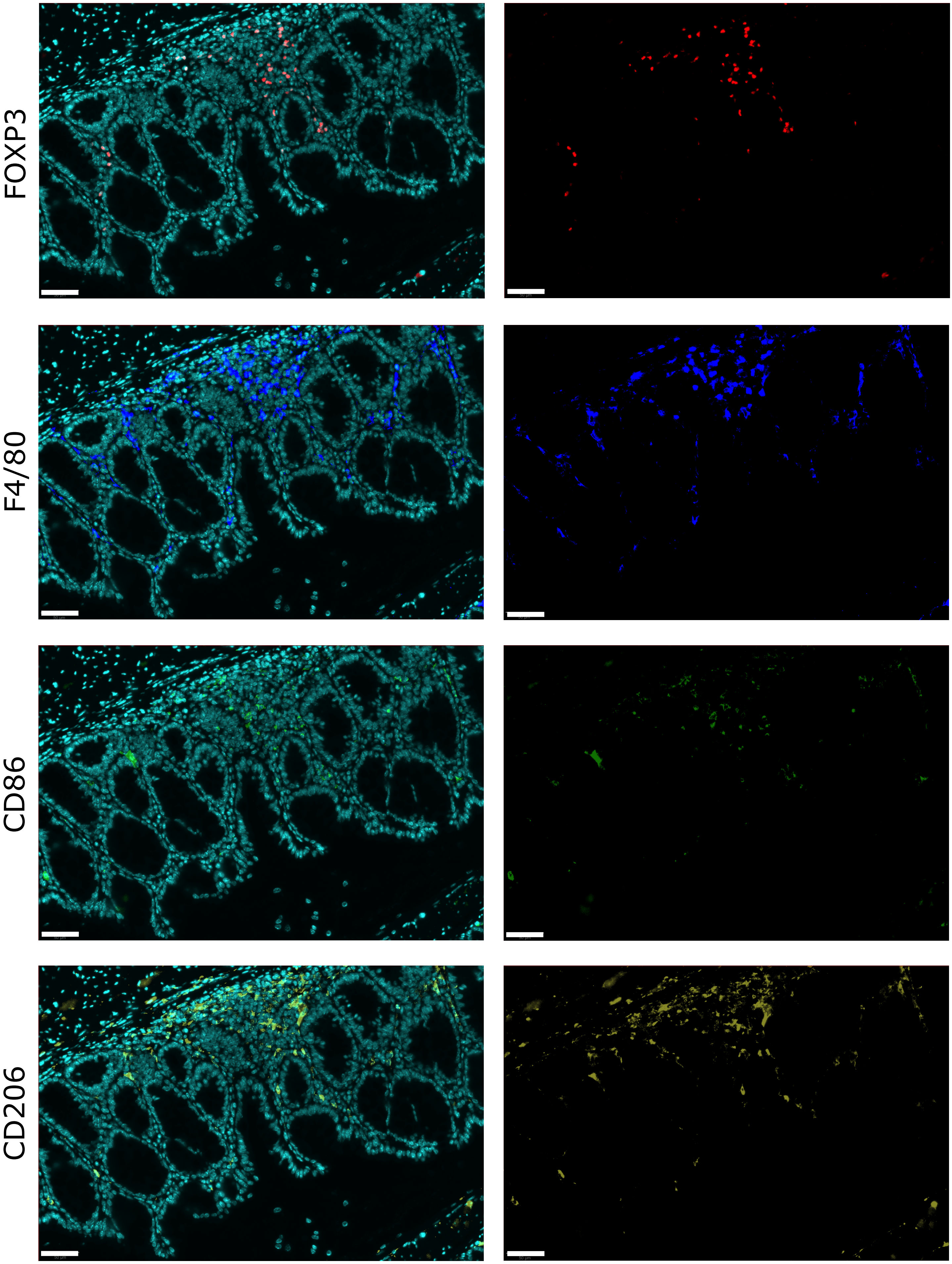
FOXP3
F4/80
CD86
CD206

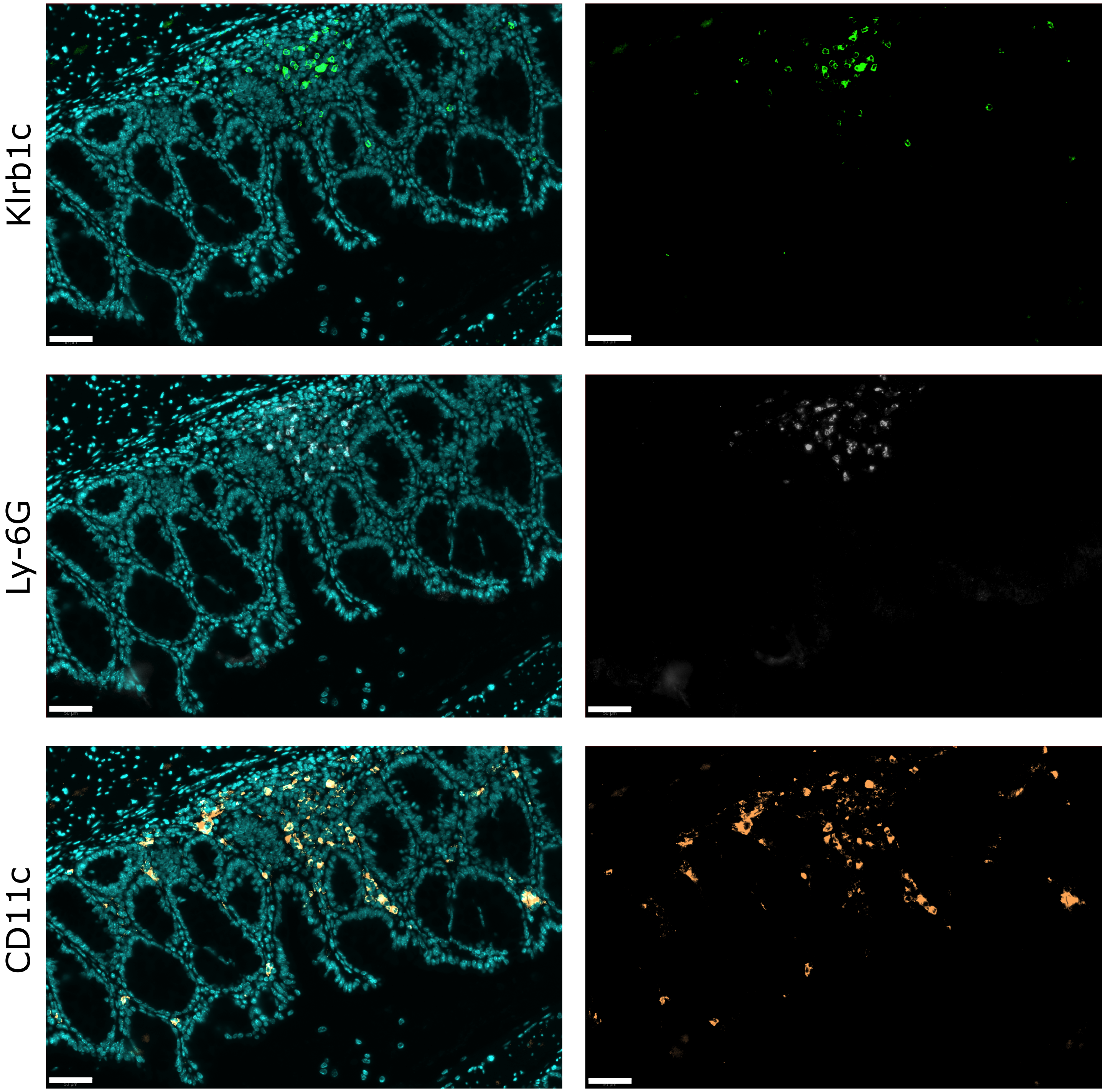

Supplementary Figure 1. Microscopic images showing representative examples of the **a)** homeostatic (vehicle-treated) and **b)** inflamed (AOM/DSS-treated) colon using H&E staining. **c)** Representative examples of IF staining generated using the COMET multiplex platform for each marker in the panel in an AOM/DSS-treated colon, with DAPI (left) and without DAPI (right): CD3 (magenta), CD4 (purple), CD8 (yellow), FOXP3 (red), F4/80 (blue), CD86 (green), CD206 (olive), Klrb1c (lime), Ly-6G (gray), and CD11c (orange). Scale bars: 100 µm (a-b), 50 µm (c).

Supplementary Figure 2

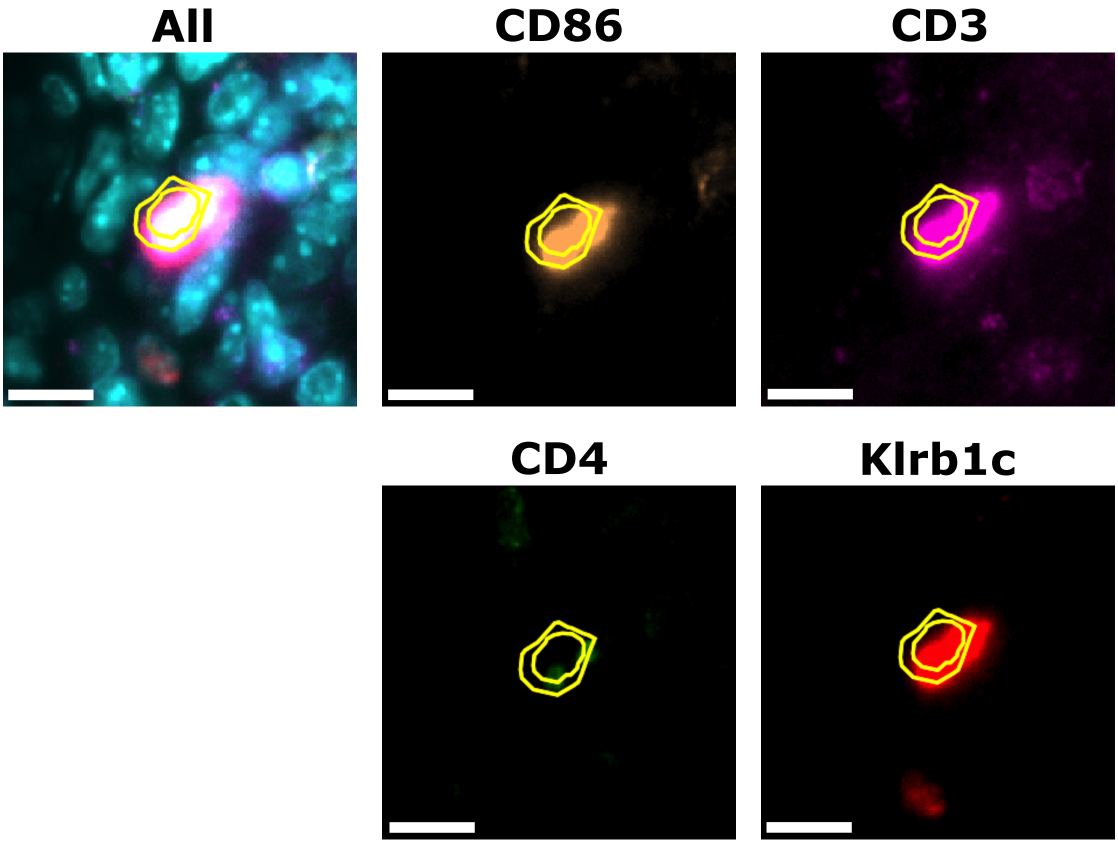

Supplementary Figure 2. Images illustrating nonspecific staining of a cell in cluster 9. Scale bars show 10 µm.

Supplementary Figure 3

a

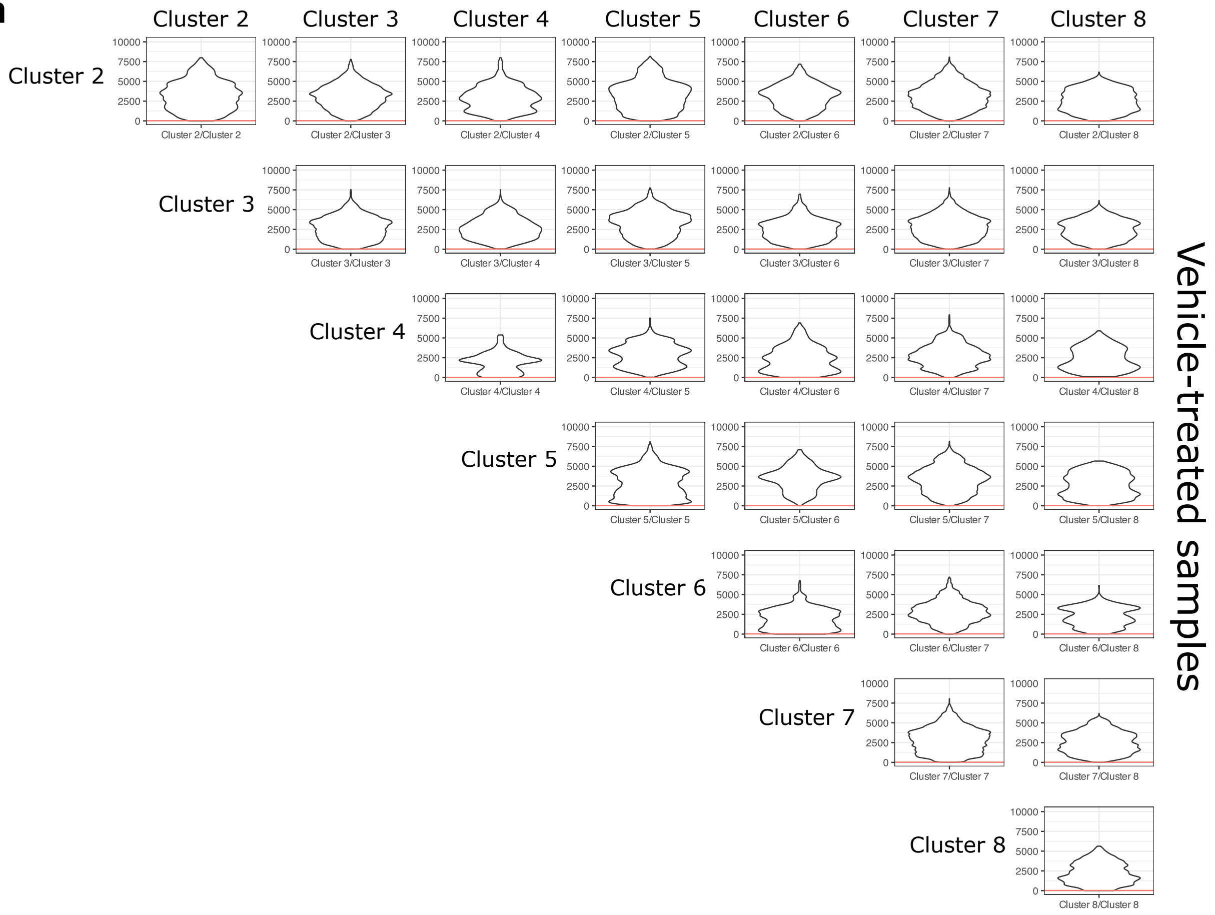

b

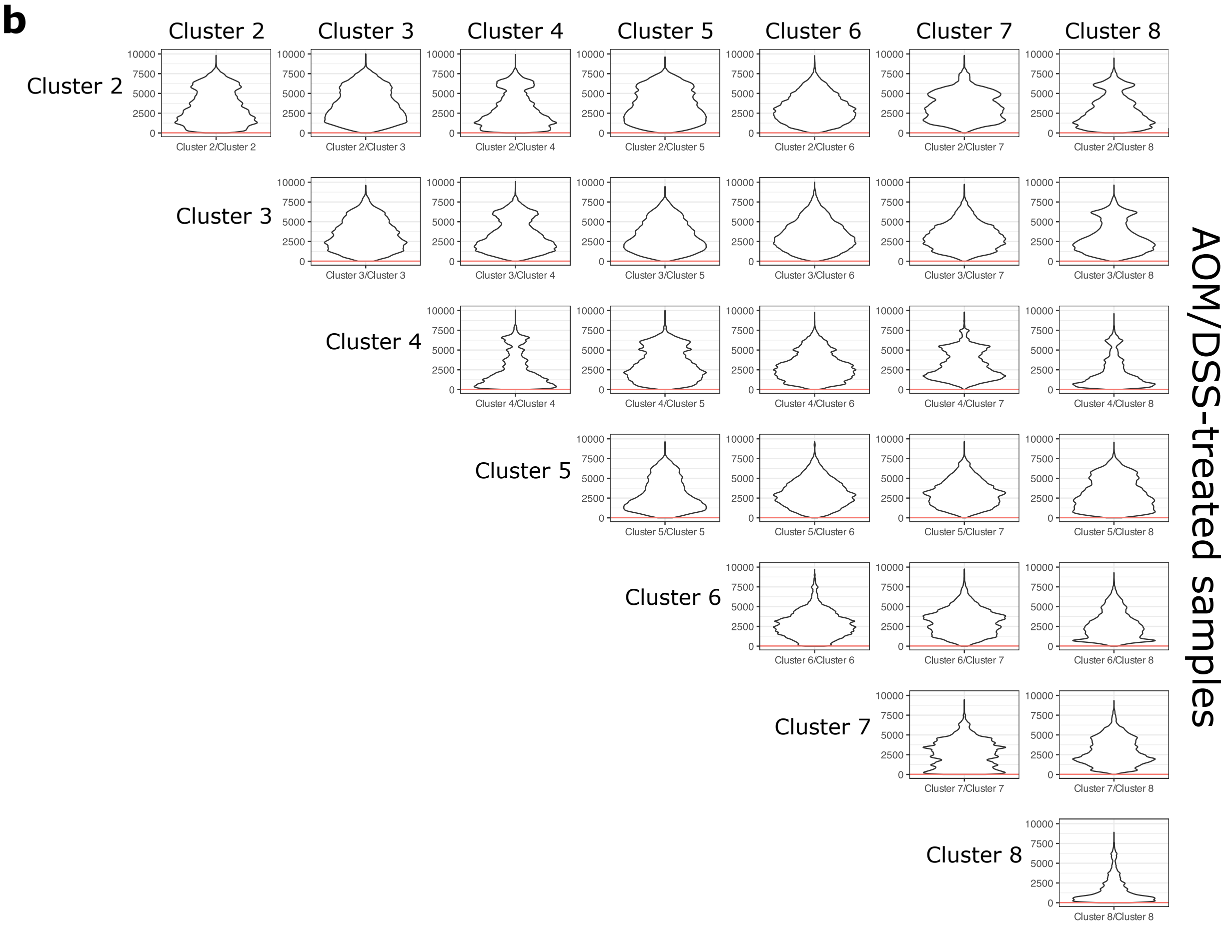

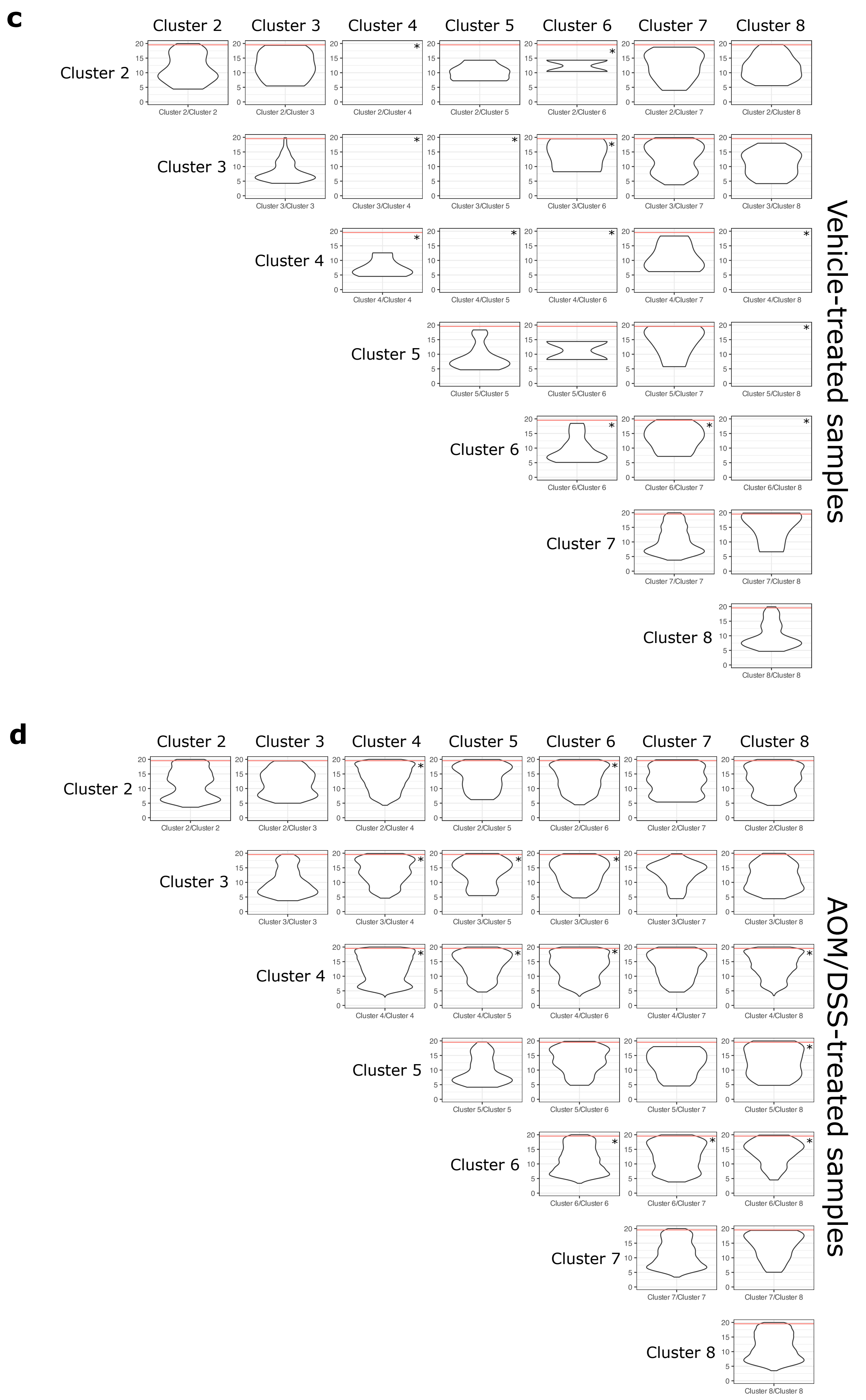

Supplementary Figure 3. Violin plots showing the distribution of cell-cell comparisons within and between clusters for all distances (range: 0 to 10,000 µm) in **a)** vehicle-treated samples and **b)** AOM/DSS-treated samples, as well as for close interactions (range: 0 to 20 µm) in **c)** vehicle-treated samples and **d)** AOM/DSS-treated samples. Y-axis represents distance (µm), and 20 µm is indicated by a red line. * indicates P<.05 between vehicle- and AOM/DSS-treated samples.

Supplementary Figure 4

a

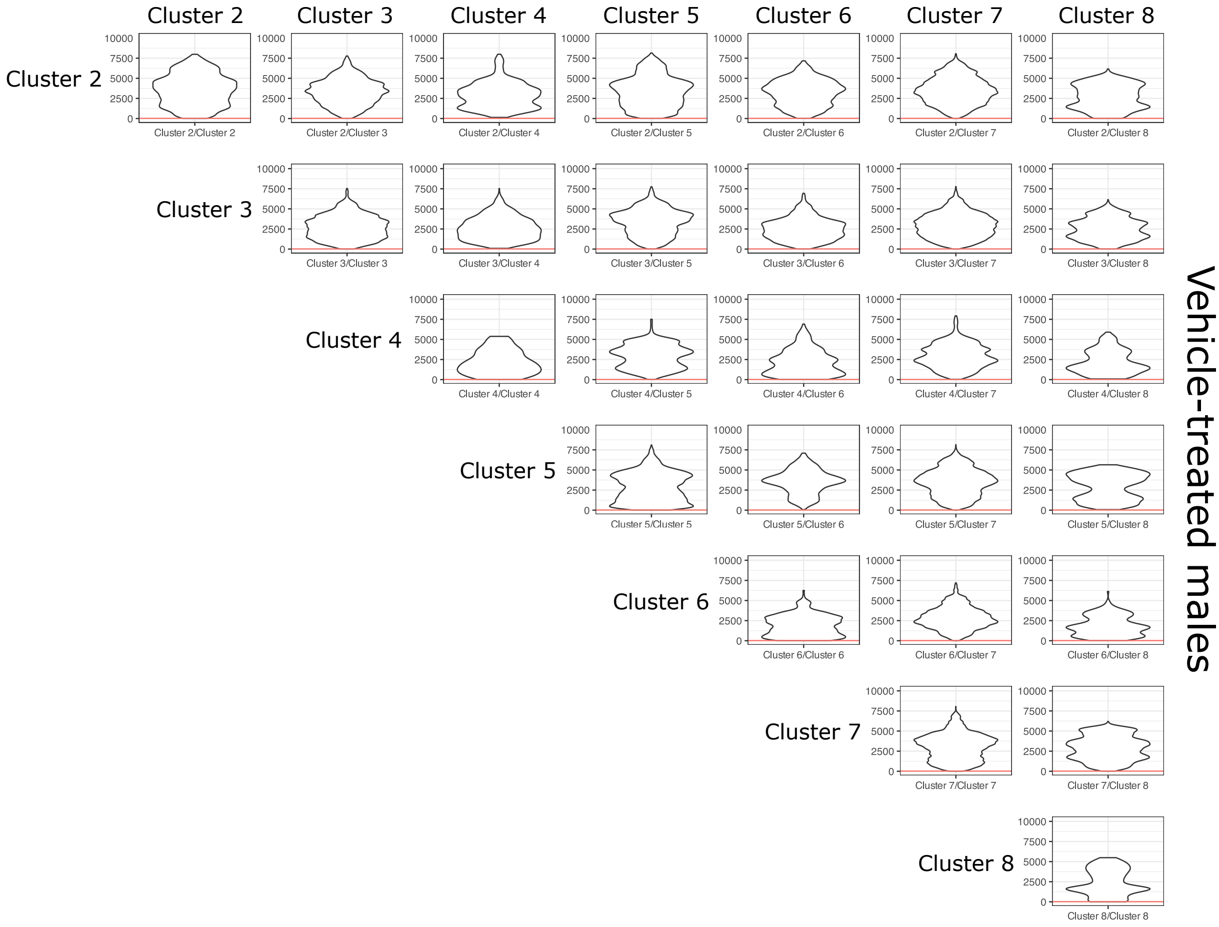

b

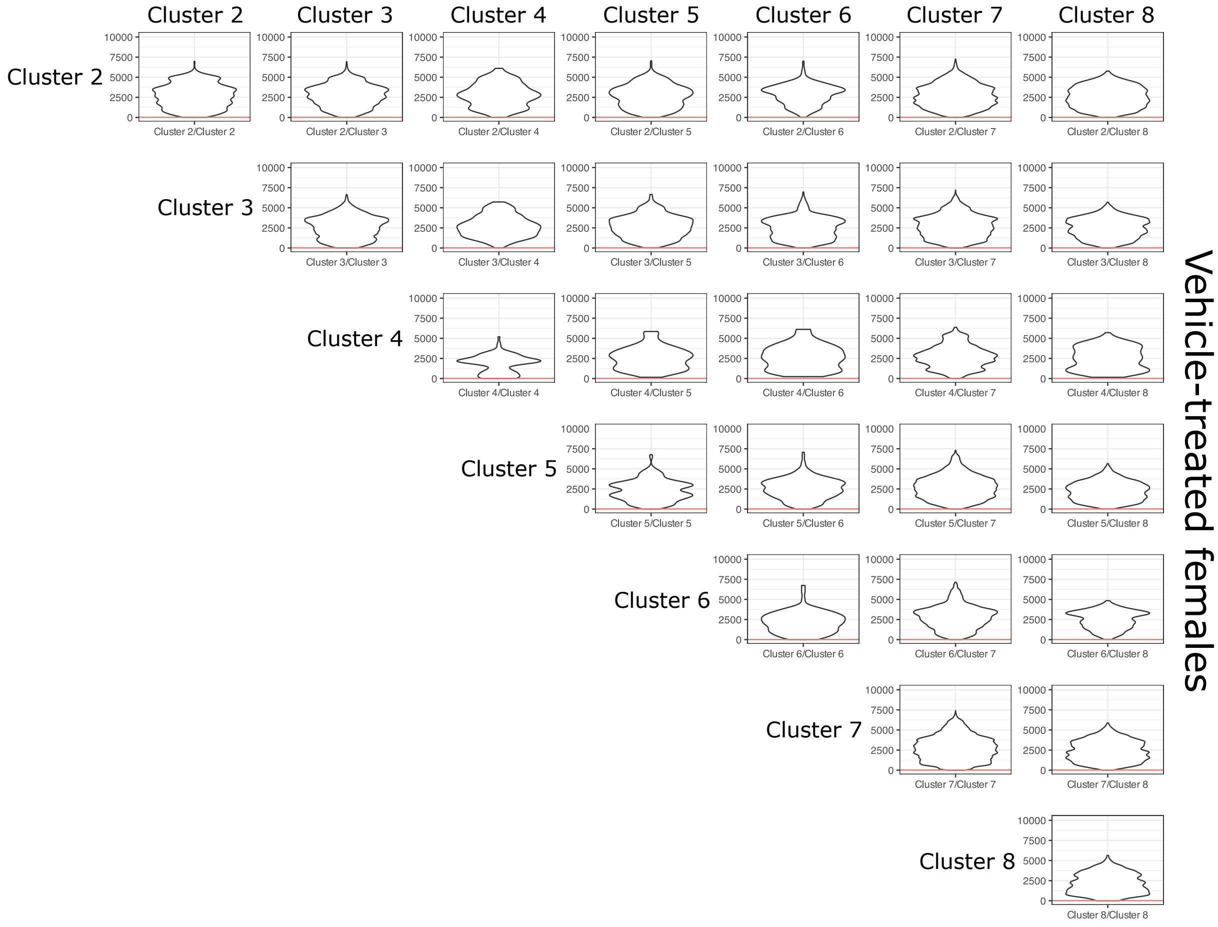

c

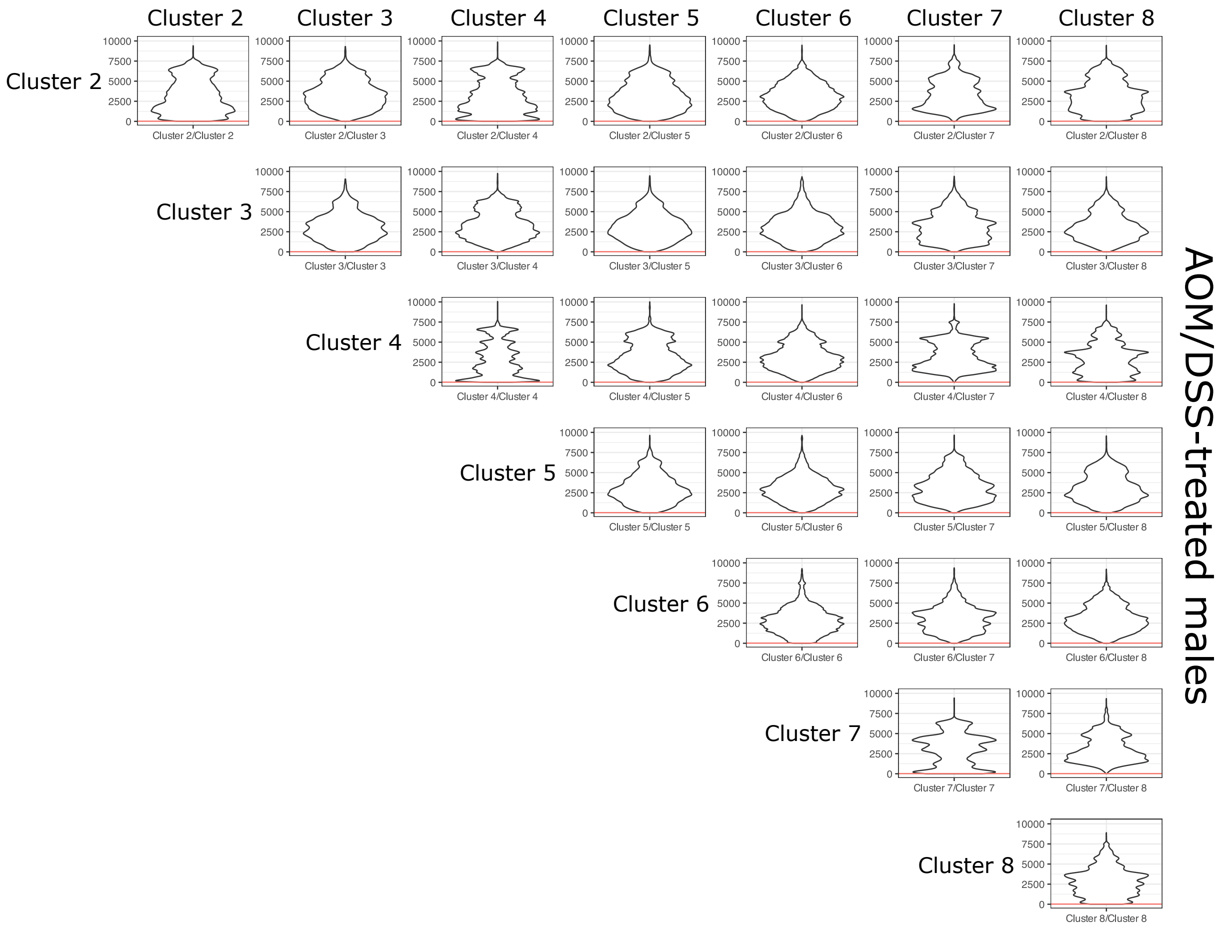

d

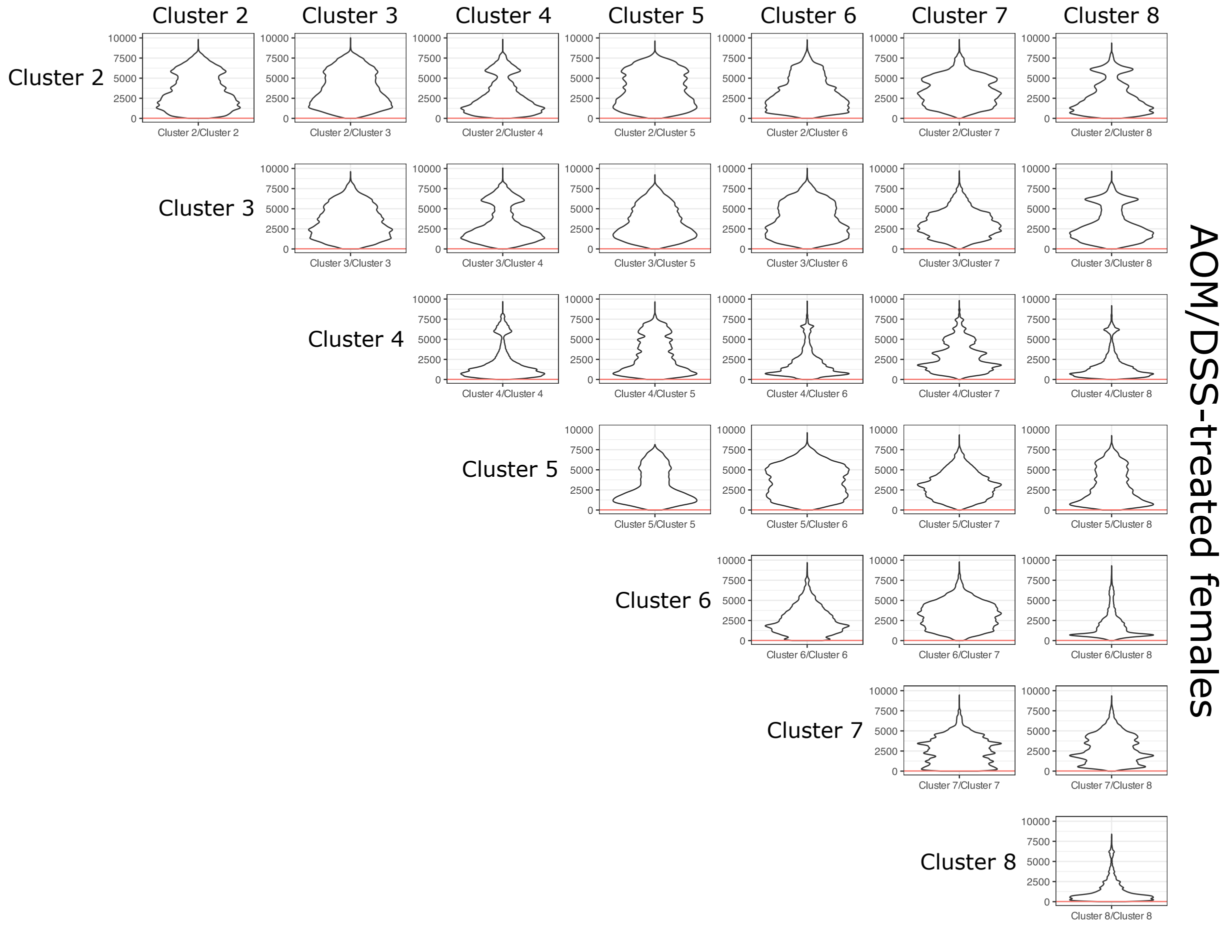

Supplementary Figure 4. Violin plots showing the distribution of all distances (range: 0 to 10,000 μm) between cell-cell comparisons within and between all clusters in **a)** vehicle-treated males, **b)** vehicle-treated females, **c)** AOM/DSS-treated males, and **d)** AOM/DSS-treated females. Y-axis represents distance (μm) and 20 μm is indicated by a red line.

Supplementary Figure 5

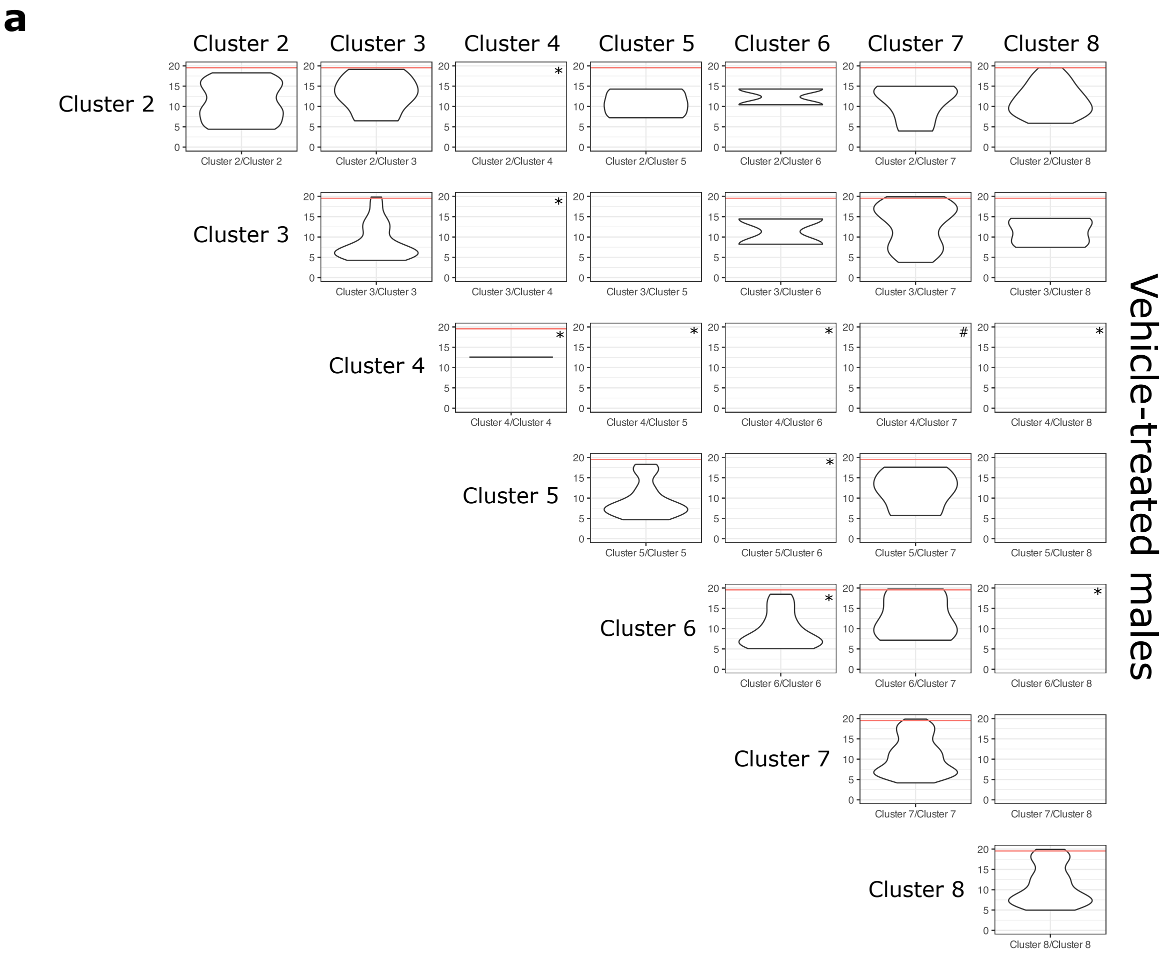

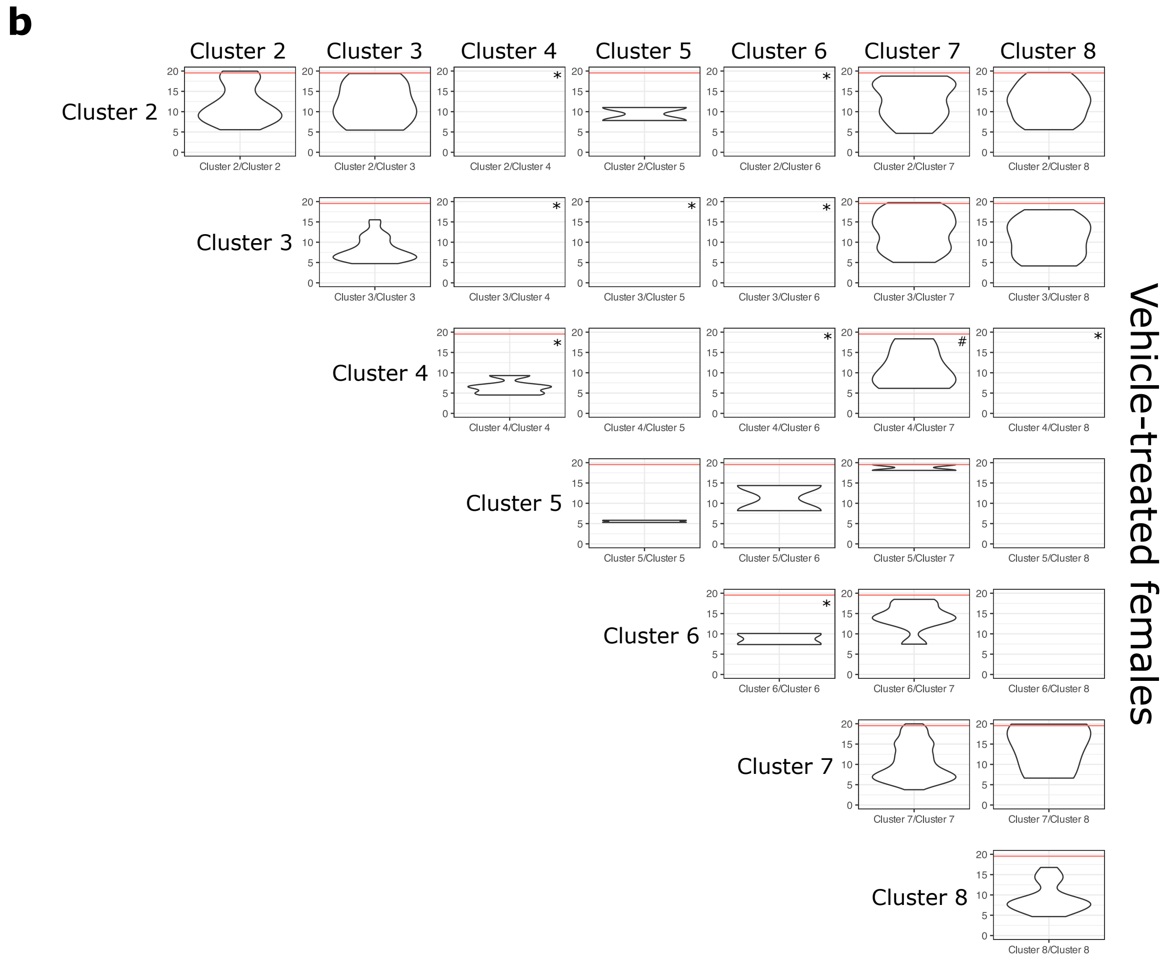

c

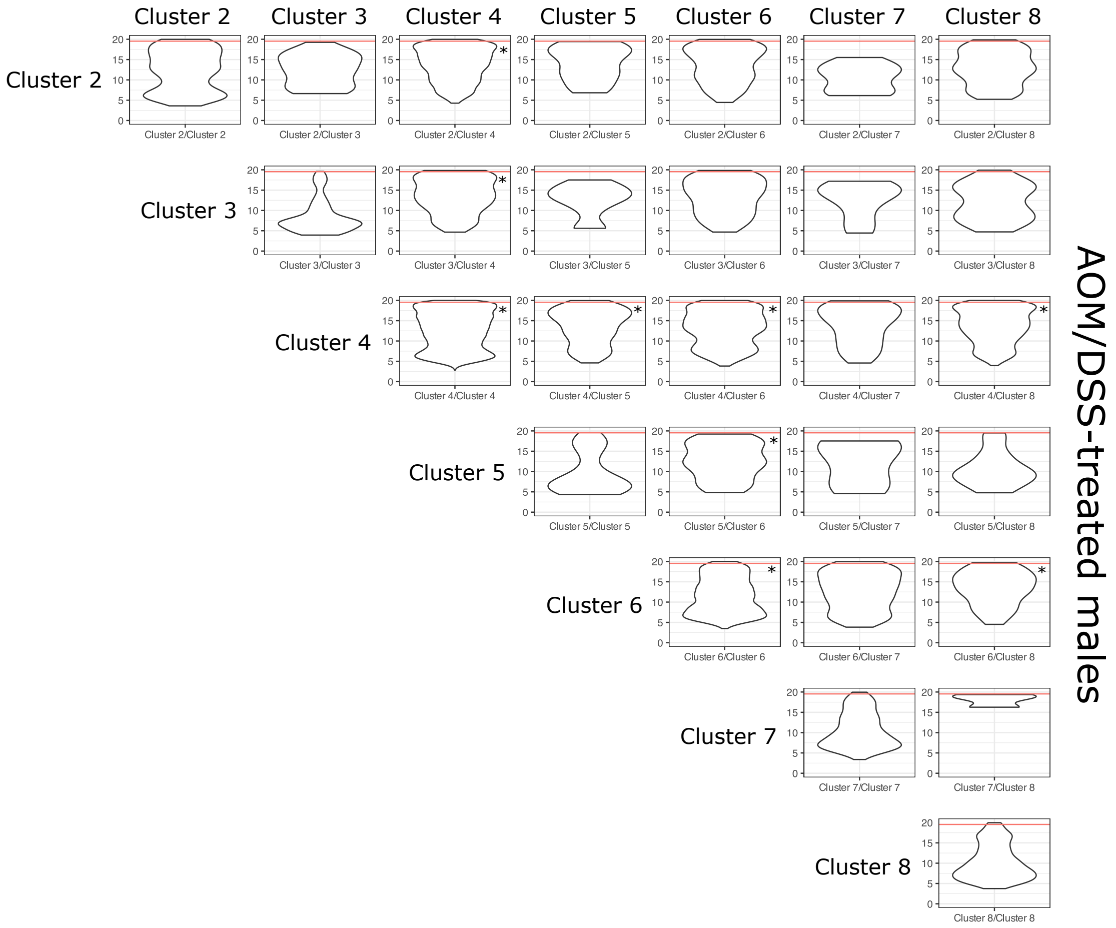

d

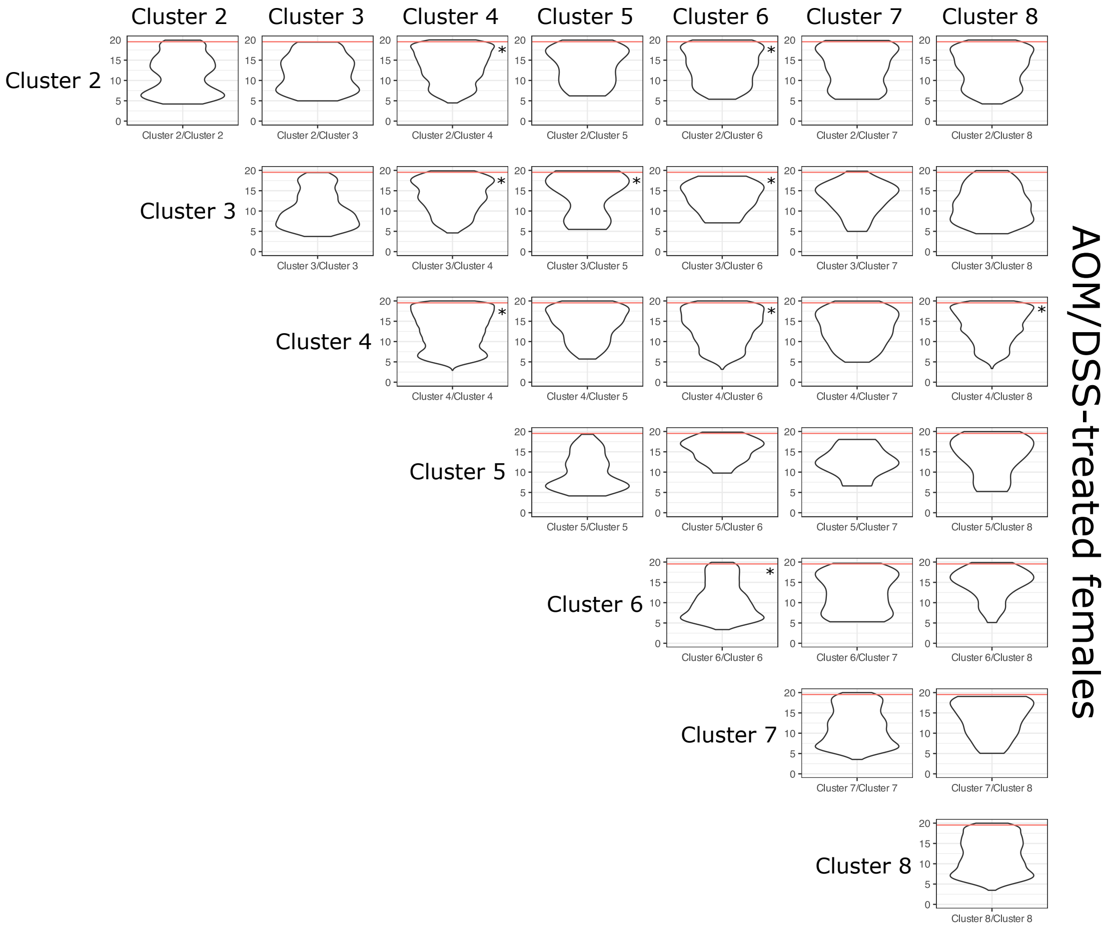

Supplementary Figure 5. Violin plots showing the distribution of distances (range: 0 to 20 μm only) between cell-cell comparisons within and between all clusters in **a)** vehicle-treated males, **b)** vehicle-treated females, **c)** AOM/DSS-treated males, and **d)** AOM/DSS-treated females. Y-axis represents distance (μm) and 20 μm is indicated by a red line. * indicates P<.05 between vehicle- and AOM/DSS-treated samples. # indicates P<.05 between vehicle-treated males and females.

Supplementary Figure 6

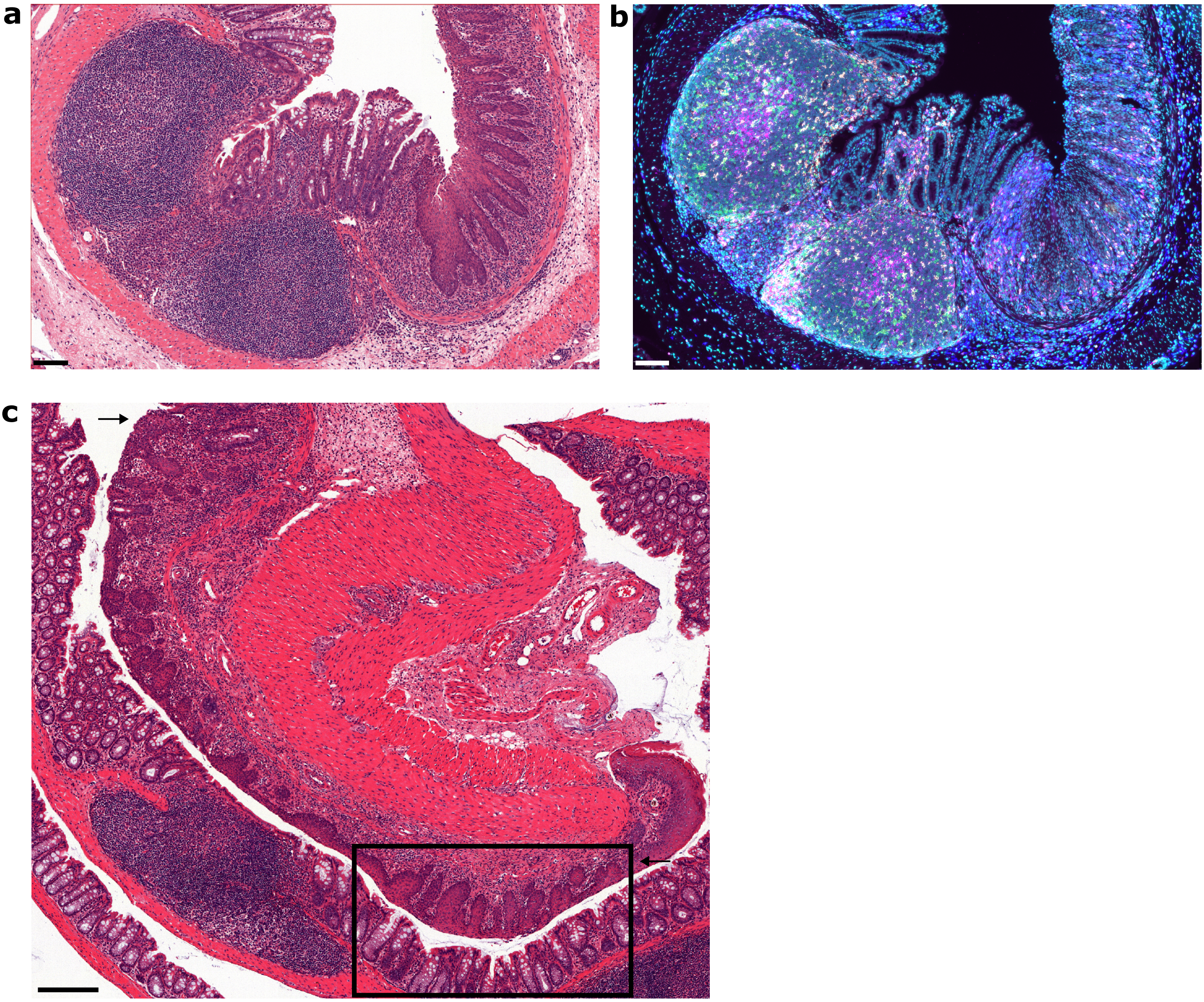

Supplementary Figure 6. Microscopic images of the colon of an AOM/DSS-treated mouse illustrating **a-b)** the isolated lymphoid follicles seen using **a)** H&E staining and **b)** multiplex immunofluorescence (CD86 (magenta), CD11c (orange), CD4 (green), and F4/80 (blue)), and **c)** a large region of squamous metaplasia using H&E staining. Arrows denote the beginning and end of the region of squamous metaplasia. The box shows the area pictured in Figure 4e. Scale bars: 100 μm (a-b), 200 μm (c).

Supplementary Figure 7

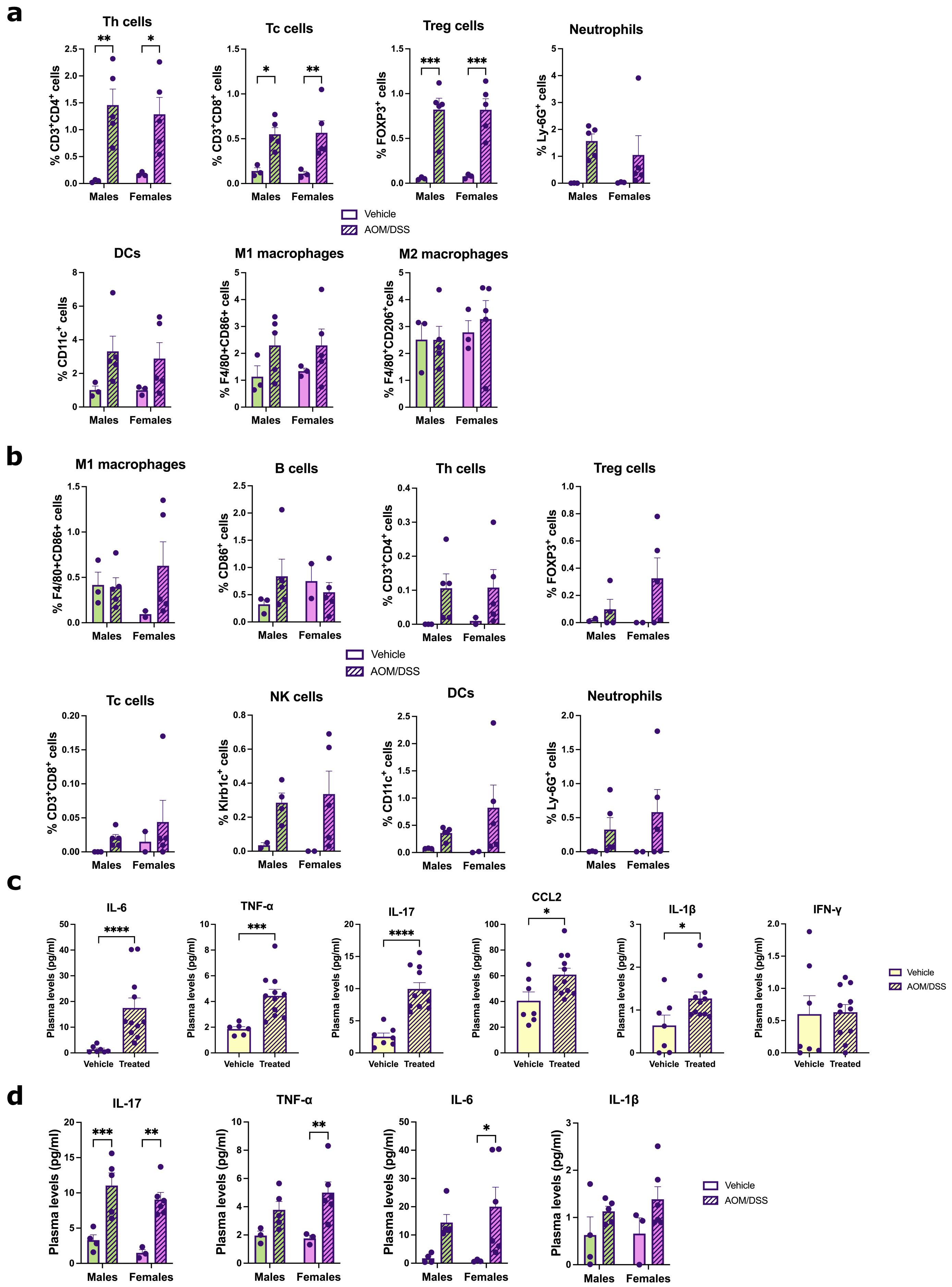

Supplementary Figure 7. a) Mucosal infiltration of Th, Tc, and Treg cells, neutrophils, DCs, and M1-like and M2-like macrophages in response to AOM/DSS treatment in males and females (n=3 vehicle-treated and n=5 AOM/DSS-treated mice of each sex). **b)** Muscular layer infiltration of all populations except M2-like macrophages in response to AOM/DSS treatment in males and females (n=2-3 vehicle-treated and n=4-5 AOM/DSS-treated mice of each sex). **c)** Plasma levels of IL-6, TNF-α, IL-17, CCL2, IL-1β, and IFNγ in mice treated with vehicle or AOM/DSS for 9 weeks (n=6-7 vehicle-treated and n=11 AOM/DSS-treated). **d)** Plasma levels of IL-17, TNFα, IL-6, and IL-1β in males and females treated with vehicle (n=4 males and n=3 females) or AOM/DSS (n=5 males and n=6 females) for 9 weeks. * indicates P<.05, ** P<.01, *** P<.001, and **** P<.0001.

Supplementary Figure 8

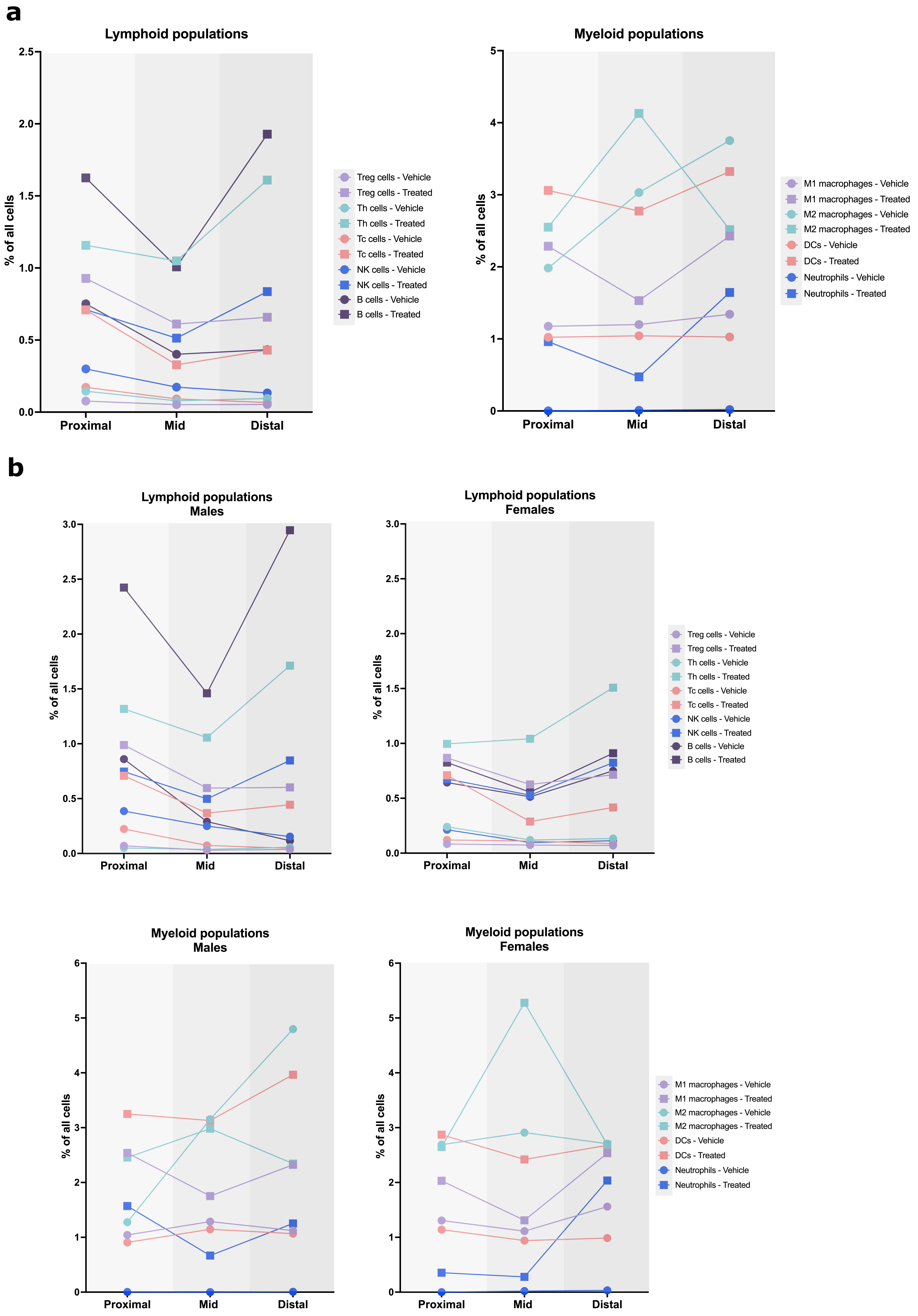

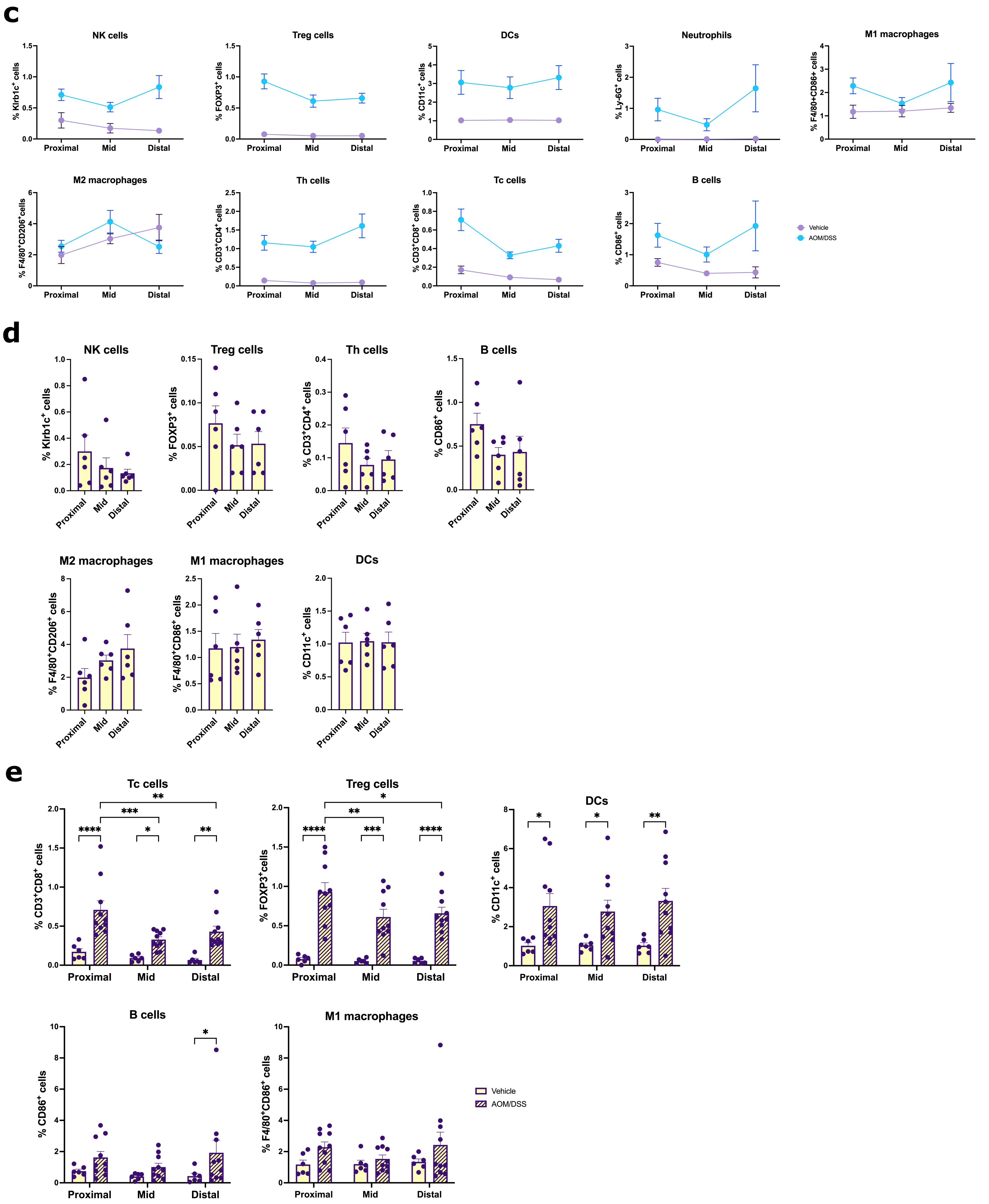

Supplementary Figure 8. Line graphs visualizing the mucosal infiltration of immune cell populations over the proximal-distal axis in vehicle- and AOM/DSS-treated mice separated by **a)** lymphoid and myeloid populations for both sexes combined (n=6 vehicle-treated and n=10 AOM/DSS-treated mice), **b)** sex (n=3 vehicle-treated and n=5 AOM/DSS-treated mice per sex), and **c)** immune cell type (n=6 vehicle-treated and n=10 AOM/DSS-treated mice, sexes combined). **d)** Mucosal infiltration of NK cells, Treg and Th cells, B cells, M2- and M1-like macrophages, and DCs in the proximal, mid, and distal colon in vehicle-treated mice (n=6, sexes combined). **e)** Mucosal infiltration of Tc and Treg cells, DCs, B cells, and M1-like macrophages in the proximal, mid, and distal colon in response to AOM/DSS treatment (n=6 vehicle-treated and n=10 AOM/DSS-treated mice, sexes combined). * indicates $P<.05$, ** $P<.01$, *** $P<.001$, and **** $P<.0001$.

Supplementary Figure 9

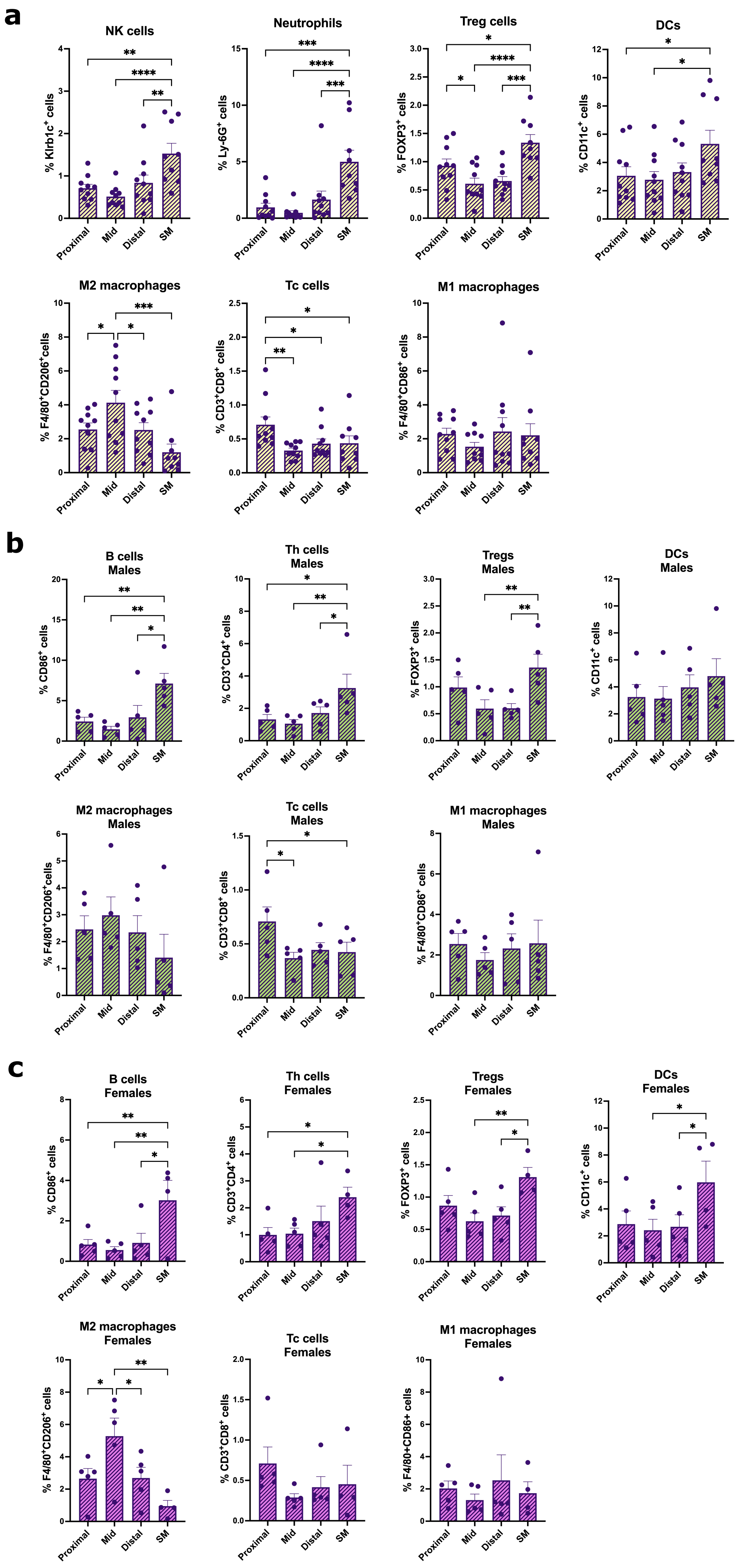

d

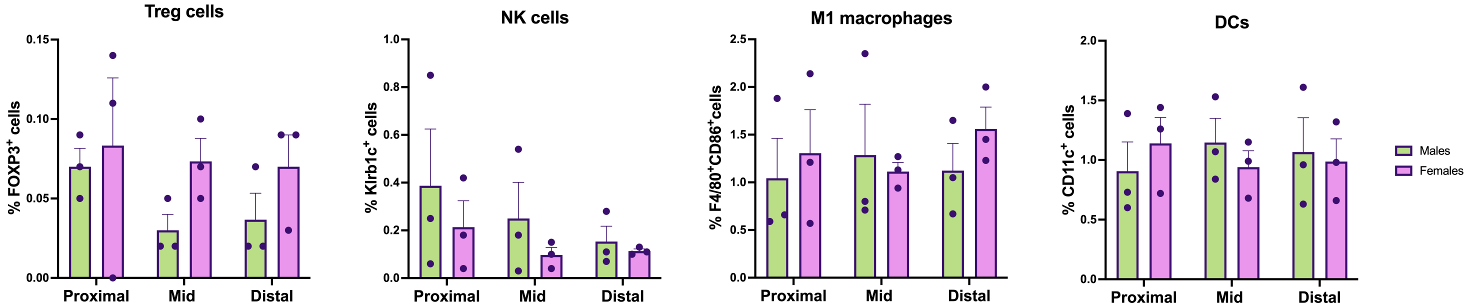

e

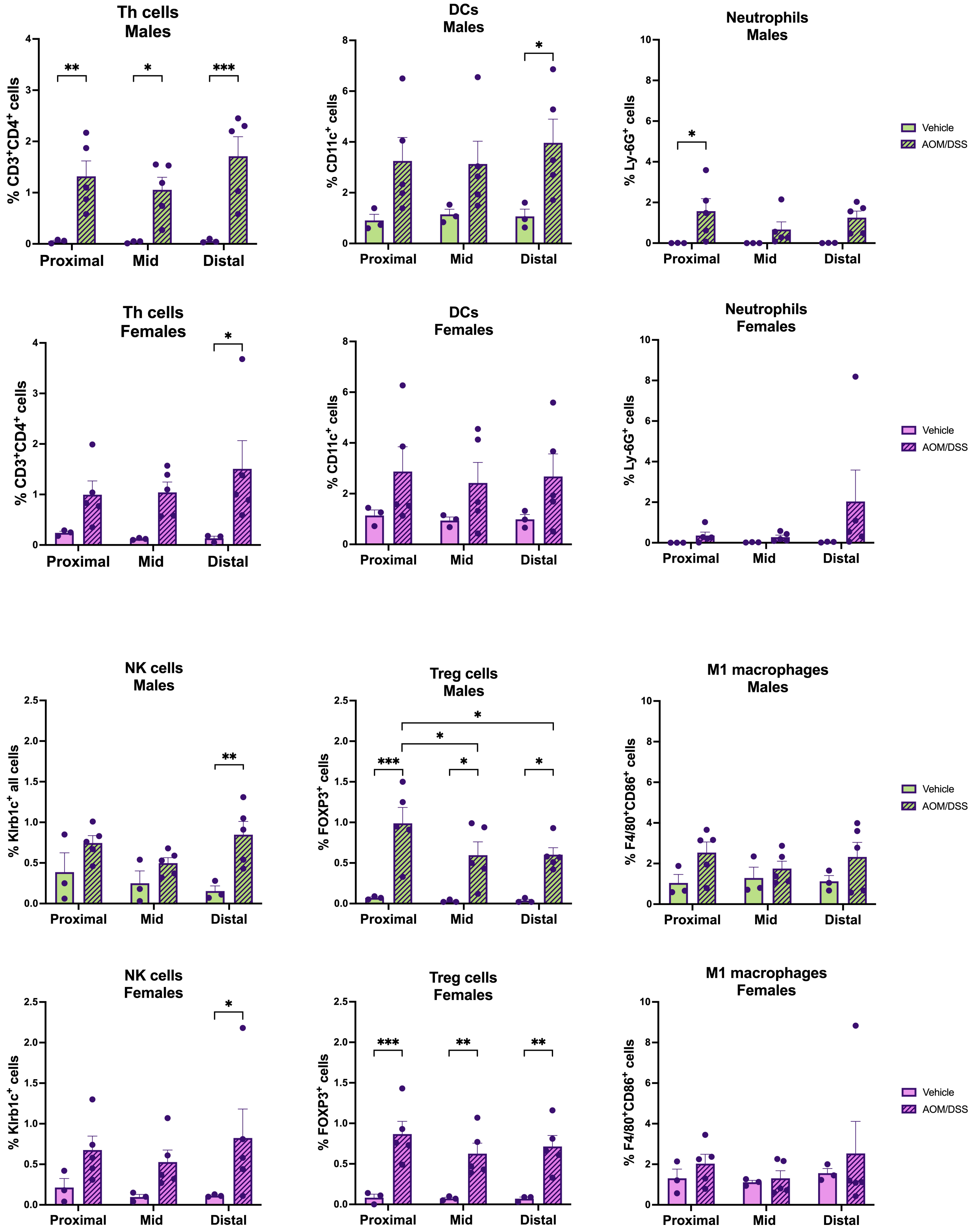

Supplementary Figure 9. **a-c)** Immune cell infiltration in regions of squamous metaplasia compared to in the proximal, mid, and distal colon in **a)** all AOM/DSS-treated samples (n=9-10, sexes combined), **b)** AOM/DSS-treated males (n=5), and **c)** AOM/DSS-treated females (n=4-5). SM=squamous metaplasia. **d)** Mucosal infiltration of Treg cells, NK cells, M1-like macrophages, and DCs in the proximal, mid, and distal colon in vehicle-treated males and females (n=3 per sex). **e)** Mucosal infiltration of Th cells, DCs, neutrophils, NK cells, Treg cells, and M1-like macrophages in response to AOM/DSS treatment in males (green, n=3) and females (purple, n=3). * indicates P<.05, ** P<.01, *** P<.001, and **** P<.0001.

Supplementary Figure 10

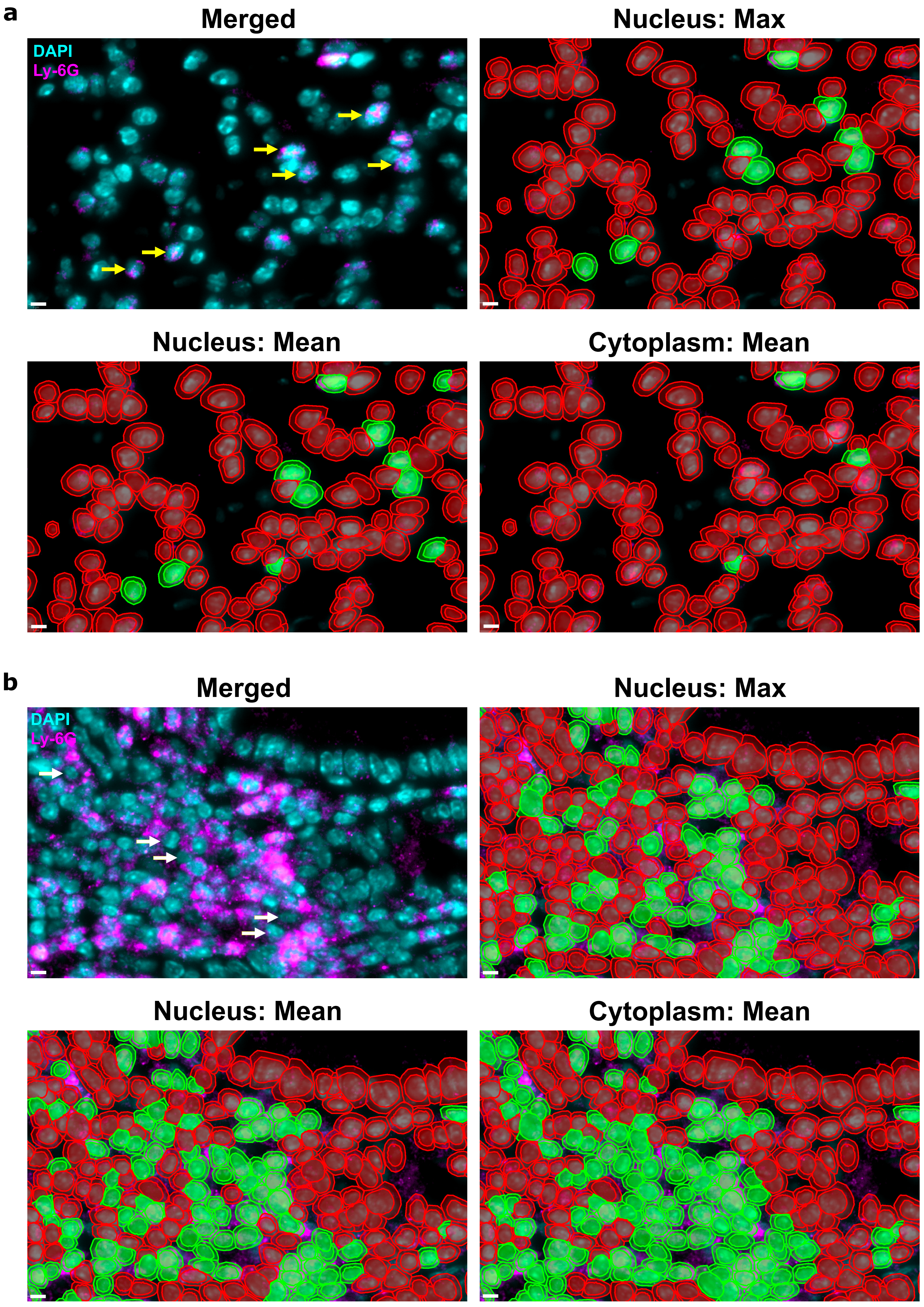

c

Classifier setting			
	**Nucleus: Max**	**Nucleus: Mean**	**Cytoplasm: Mean**
**Mucosa**	n=2963 cells (3.91%)	n=2947 cells (3.89%)	n=2413 cells (3.18%)
**Muscular layer**	n=266 cells (1.77%)	n=253 cells (1.69%)	n=88 cells (0.59%)

d

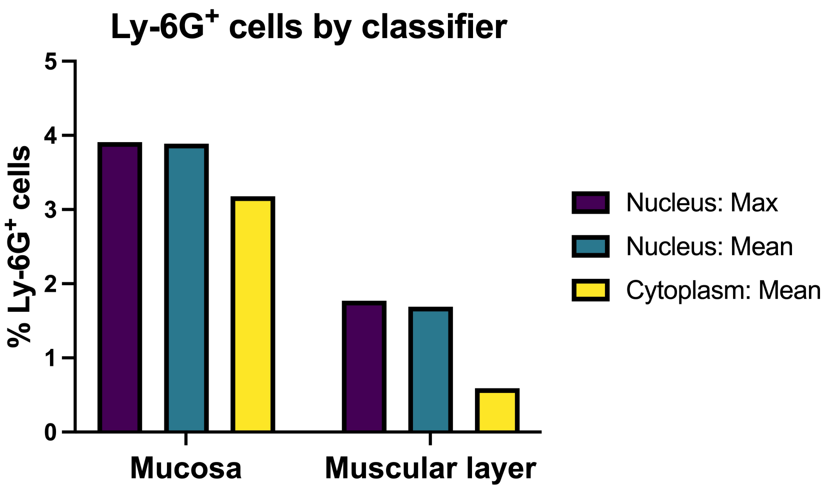

Supplementary Figure 10. a-b) Images exemplifying quantification of Ly-6G^{+} cells using three different classifier settings in two different areas of the colon. The top right panel shows the classifier setting used, while the bottom two panels show classifier settings that lead to less accurate quantification. Yellow arrows indicate examples of Ly-6G^{+} cells not identified (false negatives) using the classifier setting "Cytoplasm: Mean" (a). White arrows show examples of cells erroneously identified as Ly-6G^{+} (false positives) using the classifier setting "Cytoplasm: Mean" (b). The same threshold was used for the respective classifiers in a) and b). Scale bars: 5 µm. **c)** Table comparing the results of quantification of Ly-6G^{+} cells in the same sample using the different classifier settings. **d)** Graph comparing the percentage of Ly-6G^{+} cells quantified using the three different classifier settings.

Supplementary Figure 11

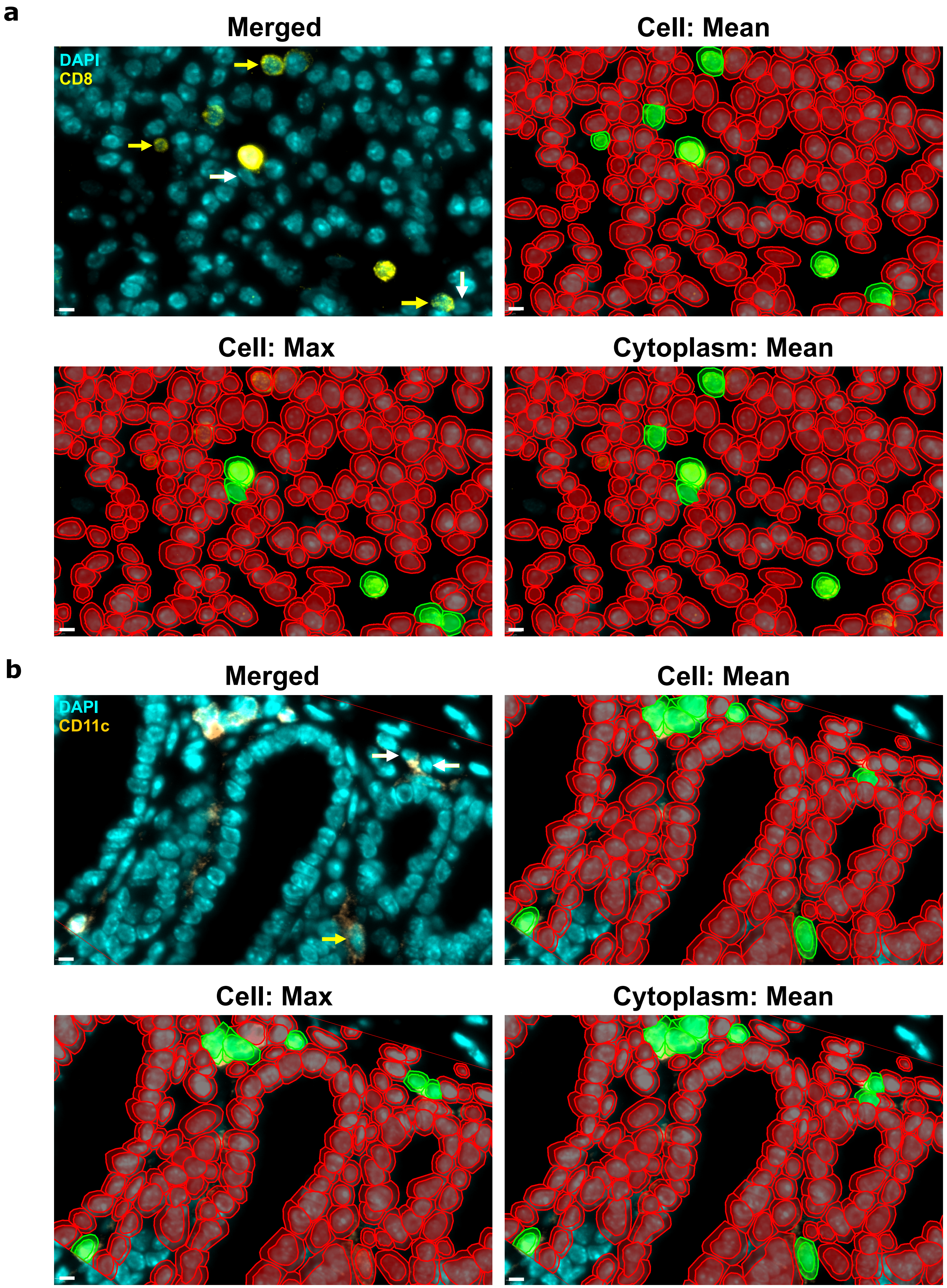

Supplementary Figure 11. Images exemplifying quantification of **a)** CD8$^+$ cells and **b)** CD11c$^+$ cells using three different classifier settings. The top right image in each panel shows the classifier setting used, while the bottom two panels show classifier settings that lead to less accurate quantification. Yellow arrows indicate examples of positive cells not identified by either the classifier setting "Cell: Max" or "Cytoplasm: Mean". White arrows indicate examples of cells erroneously identified as positive by either the classifier setting "Cell: Max" or "Cytoplasm: Mean". Scale bars: 5 μm.
