# Supplementary material for: Spatial profiling of the mouse colonic immune landscape associated with colitis and sex
Source: Commun Biol. 2024 Nov 29;7:1595. doi: 10.1038/s42003-024-07276-1 (PMC11606951; doi:10.1038/s42003-024-07276-1)
Supplement: Supplementary file 2 — Description of Additional Supplementary Files [file 42003_2024_7276_MOESM2_ESM.pdf]

Description of Additional Supplementary Files

File name: Supplementary Data 1

Description: The number of cell-cell comparisons in each cluster/cluster comparison for all violin plots (all distances and range 0-20 μm) in vehicle- and AOM/DSS-treated samples.

File name: Supplementary Data 2

Description: The number of cell-cell comparisons in each cluster/cluster comparison for all violin plots (all distances and range 0-20 μm) when samples were stratified by sex.

File name: Supplementary Data 3

Description: COMET parameters.

File name: Supplementary Data 4

Description: The source data behind the graphs in the paper
