# Supplementary material for: Spatial profiling of the mouse colonic immune landscape associated with colitis and sex
Source: Commun Biol. 2024 Nov 29;7:1595. doi: 10.1038/s42003-024-07276-1 (PMC11606951; doi:10.1038/s42003-024-07276-1)
Supplement: Supplementary file 7 — Reporting summary [file 42003_2024_7276_MOESM7_ESM.pdf]

nature portfolio

Corresponding author(s): Cecilia Williams
Last updated by author(s): Oct 23, 2024

Reporting Summary

Nature Portfolio wishes to improve the reproducibility of the work that we publish. This form provides structure for consistency and transparency in reporting. For further information on Nature Portfolio policies, see our Editorial Policies and the Editorial Policy Checklist.

Statistics

For all statistical analyses, confirm that the following items are present in the figure legend, table legend, main text, or Methods section.

n/a	Confirmed	
☐	☒	The exact sample size (*n*) for each experimental group/condition, given as a discrete number and unit of measurement
☐	☒	A statement on whether measurements were taken from distinct samples or whether the same sample was measured repeatedly
☐	☒	The statistical test(s) used AND whether they are one- or two-sided<br>*Only common tests should be described solely by name; describe more complex techniques in the Methods section.*
☒	☐	A description of all covariates tested
☐	☒	A description of any assumptions or corrections, such as tests of normality and adjustment for multiple comparisons
☐	☒	A full description of the statistical parameters including central tendency (e.g. means) or other basic estimates (e.g. regression coefficient) AND variation (e.g. standard deviation) or associated estimates of uncertainty (e.g. confidence intervals)
☐	☒	For null hypothesis testing, the test statistic (e.g. *F*, *t*, *r*) with confidence intervals, effect sizes, degrees of freedom and *P* value noted<br>*Give P values as exact values whenever suitable.*
☒	☐	For Bayesian analysis, information on the choice of priors and Markov chain Monte Carlo settings
☒	☐	For hierarchical and complex designs, identification of the appropriate level for tests and full reporting of outcomes
☒	☐	Estimates of effect sizes (e.g. Cohen's *d*, Pearson's *r*), indicating how they were calculated

Our web collection on statistics for biologists contains articles on many of the points above.

Software and code

Policy information about availability of computer code

Data collection	The StarDist extension (publicly available online) for QuPath was used for nuclear segmentation to quantify cells.
Data analysis	The Spatial Image Analysis of Tissues (SPIAT) R package used in this study is publicly available online. The modified scripts used in our study have been deposited in GitHub and made public.

For manuscripts utilizing custom algorithms or software that are central to the research but not yet described in published literature, software must be made available to editors and reviewers. We strongly encourage code deposition in a community repository (e.g. GitHub). See the Nature Portfolio guidelines for submitting code & software for further information.

Data

Policy information about availability of data

All manuscripts must include a data availability statement. This statement should provide the following information, where applicable:

- Accession codes, unique identifiers, or web links for publicly available datasets
- A description of any restrictions on data availability
- For clinical datasets or third party data, please ensure that the statement adheres to our policy

The scripts for the clustering, dimensionality reduction, and spatial analysis can be found at https://github.com/Linastep-bot/spatial-profiling-of-colonic-immune-microenvironment

Research involving human participants, their data, or biological material

Policy information about studies with human participants or human data. See also policy information about sex, gender (identity/presentation), and sexual orientation and race, ethnicity and racism.

Reporting on sex and gender	N/A
Reporting on race, ethnicity, or other socially relevant groupings	N/A
Population characteristics	N/A
Recruitment	N/A
Ethics oversight	N/A

Note that full information on the approval of the study protocol must also be provided in the manuscript.

Field-specific reporting

Please select the one below that is the best fit for your research. If you are not sure, read the appropriate sections before making your selection.

☒ Life sciences ☐ Behavioural & social sciences ☐ Ecological, evolutionary & environmental sciences

For a reference copy of the document with all sections, see nature.com/documents/nr-reporting-summary-flat.pdf

Life sciences study design

All studies must disclose on these points even when the disclosure is negative.

Sample size	Sample size was estimated prior to the implementation of the experiment to ensure statistical power of detection. Sample size is reported as a number where relevant.
Data exclusions	For clustering analysis, one sample was excluded as it failed to normalize properly. During manual annotation for quantification of immune cell infiltration, areas with nonspecific staining, dust, edge effect, or areas that were torn were excluded.
Replication	The antibody panel used for the multiplex staining was thoroughly optimized and all antibodies included in the final panel were rigorously tested for specific staining and sufficient staining intensity to ensure reproducibility.
Randomization	A statement regarding randomization of mice is provided in the "Material and methods" section under the subheading "In vivo experiments". In summary, mice of both sexes were randomly assigned to treatment with vehicle or AOM/DSS.
Blinding	Investigators were blinded to data analysis as manual quantification of immune cell numbers was performed blinded to sample treatment, genotype, and sex.

Reporting for specific materials, systems and methods

We require information from authors about some types of materials, experimental systems and methods used in many studies. Here, indicate whether each material, system or method listed is relevant to your study. If you are not sure if a list item applies to your research, read the appropriate section before selecting a response.

Materials & experimental systems

n/a	Involved in the study	
☐	☒	Antibodies
☒	☐	Eukaryotic cell lines
☒	☐	Palaeontology and archaeology
☐	☒	Animals and other organisms
☒	☐	Clinical data
☒	☐	Dual use research of concern
☒	☐	Plants

Methods

n/a	Involved in the study	
☒	☐	ChIP-seq
☒	☐	Flow cytometry
☒	☐	MRI-based neuroimaging

Antibodies

Antibodies used	The full list of antibodies used is provided in the manuscript in Table 1, which contains all relevant details.
Validation	All antibodies in the final panel used for multiplex staining were first validated manually by single-plex immunofluorescence, prior to inclusion in the multiplex panel. Antibodies that did not show any specific staining were excluded and all antibodies were rigorously tested and dilutions were also optimized to ensure specificity and sufficiently strong staining.

Animals and other research organisms

Policy information about studies involving animals; ARRIVE guidelines recommended for reporting animal research, and Sex and Gender in Research

Laboratory animals	Five- to ten-week-old mice of both sexes maintained on a C57BL/6J background.
Wild animals	The study did not involve wild animals.
Reporting on sex	Sex was considered in the study design, as we were interested in studying sex differences and therefore included a sufficient number of mice of each sex in each group to ensure statistical power. n=3 vehicle-treated mice and n=5 AOM/DSS-treated mice of each sex were used in this study. Sex-based analyses are reported where performed in the manuscript.
Field-collected samples	The study did not involve samples collected from the field.
Ethics oversight	This study and all experimental protocols were approved by the local ethical committee of the Swedish National Board of Animal Research (Approval ID: 211/16).

Note that full information on the approval of the study protocol must also be provided in the manuscript.

Plants

Seed stocks	N/A
Novel plant genotypes	N/A
Authentication	N/A
